# O-Antigen Gene Clusters of *Plesiomonas shigelloides* Serogroups and Its Application in Development of a Molecular Serotyping Scheme

**DOI:** 10.3389/fmicb.2019.00741

**Published:** 2019-04-10

**Authors:** Daoyi Xi, Xiaochen Wang, Kexin Ning, Qian Liu, Fuyi Jing, Xi Guo, Boyang Cao

**Affiliations:** ^1^Key Laboratory of Molecular Microbiology and Technology of the Ministry of Education, TEDA College, Nankai University, Tianjin, China; ^2^TEDA Institute of Biological Sciences and Biotechnology, Nankai University, Tianjin, China; ^3^Tianjin Research Center for Functional Genomics and Biochips, TEDA College, Nankai University, Tianjin, China; ^4^Tianjin Key Laboratory of Microbial Functional Genomics, TEDA College, Nankai University, Tianjin, China

**Keywords:** *Plesiomonas shigelloides*, O-antigen gene clusters, serogroups, PCR, luminex-based array

## Abstract

*Plesiomonas shigelloides* is a Gram-negative, flagellated, rod-shaped, ubiquitous, and facultative anaerobic bacterium. It has been isolated from various sources, such as freshwater, surface water, and many wild and domestic animals. *P. shigelloides* is associated with diarrheal diseases of acute secretory gastroenteritis, an invasive shigellosis-like disease, and a cholera-like illness in humans. At present, 102 somatic antigens and 51 flagellar antigens of *P. shigelloides* have been recognized; however, very little is known about variations of O-antigens among *P. shigelloides* species. In this study, 12 O-antigen gene clusters of *P. shigelloides*, O2H1a1c (G5877), O10H41 (G5892), O12H35 (G5890), O23H1a1c (G5263), O25H3 (G5879), O26H1a1c (G5889), O32H37 (G5880), O33H38 (G5881), O34H34 (G5882), O66H3 (G5270), O75H34 (G5885), and O76H39 (G5886), were sequenced and analyzed. The genes that control O-antigen synthesis are present as chromosomal gene clusters that maps between *rep* and *aqpZ*, and most of the synthesis and translocation of OPS (O-specific polysaccharide) belongs to Wzx/Wzy pathway with the exception of O12, O25, and O66, which use the ATP-binding cassette (ABC) transporter pathway. Phylogenetic analysis of *wzx* and *wzy* show that the *wzx* and *wzy* genes are specific to individual O-antigens and can be used as targets in molecular typing. Based on the sequence data, an O-antigen specific suspension array that detects 12 distinct OPS’ has been developed. This is the first report to catalog the genetic features of *P. shigelloides* O-antigen variations and develop a suspension array for the molecular typing. The method has several advantages over traditional bacteriophage and serum agglutination methods and lays the foundation for easier identification and detection of additional O-antigen in the future.

## Introduction

*Plesiomonas shigelloides* was first isolated from the feces of a patient for whom no clinical history was available. It was reported to be a motile organism with the major antigen of *Shigella sonnei* phase I, and originally named *Paracolon* C27 by Ferguson and Henderson (1947), (Hu et al., 2013). Biochemical, morphological, and taxonomical features of the organism were collected by many investigators and the organism was reclassified into the family *Enterobacteriaceae*, being the only oxidase-positive member of this family (Martinez-Murcia et al., 1992; Huys and Swings, 1999; Garrity et al., 2001). *P. shigelloides*, the only species of the genus, is a Gram-negative, flagellated, rod-shaped, ubiquitous, and facultative anaerobic bacterium, which has been later isolated from various sources, such as freshwater, surface water, and many wild and domestic animals (Wong and Pang, 2000). *P. shigelloides* is associated with diarrheal diseases in humans (Brenden et al., 1988), including acute secretory gastroenteritis (Mandal et al., 1982), an invasive shigellosis-like disease (McNeeley et al., 1984), and a cholera-like illness (Tsukamoto et al., 1978). Traveler diarrhea affects millions of tourists each year. *P. shigelloides* has been ranked third in traveler’s diarrhea in Japan (Schubert and Holz-Bremer, 1999), and third as a cause of diarrhea among a number of military units in China (Bai et al., 2004), as well as among civilians in Hong Kong (Chan et al., 2003).

The O-antigen is a component of lipopolysaccharides (LPS) present in the outer membrane of Gram-negative bacteria and comprised of repeated oligosaccharide units (O-units, OPS). The OPS is the major contribution of the antigenic variability of the cell surface and is subject to intense selection by the host immune system and other environmental factors, such as bacteriophages, which may account for the existence of diverse O-antigen forms within a species (Reeves et al., 1996). The O-antigen gene clusters consist of genes that belong to three main classes: nucleotide sugar biosynthesis pathway genes, glycosyltransferase (GT) genes, and O-unit processing genes. Three distinct pathways participated in the synthesis and translocation of OPS: the Wzx/Wzy pathway, the ATP-binding cassette (ABC) transporter pathway (Hu et al., 2013), and the synthase pathway. O-antigen serotyping, targeting the *wzx* and *wzy* genes, is able to detect distinct O-antigen forms, including *E. coli*, *Shigella*, *Salmonella*, *Vibrio parahaemolyticus*, *Acinetobacter*, *Hafnia*, and *Proteus* (Li et al., 2009, 2010; Guo et al., 2013; Hu et al., 2013; Chen et al., 2015; Duan et al., 2016; Yu et al., 2017). Our previous studies employed *wzx* and *wzy* for serotyping *E. coli* and *Shigella* by microarray (Li et al., 2006), *wzx* and *wzy* for serotyping *Pseudomonas aeruginosa* (Li et al., 2018) and *wzm* and *wzt* for serotyping 15 *Legionella pneumophila* serogroups by microarray (Cao et al., 2013). In this report, *wzx, wzy*, and *wzm* have been screened and used as targets for molecular serotyping of the 12 *P. shigelloides* strains.

At present, 102 somatic antigens and 51 flagellar antigens of *P. shigelloides* have been recognized (Aldova and Shimada, 2000). *P. shigelloides* strain 302-73, isolated in Japan (Shimada and Sakazaki, 1985), is the representative strain for the O1 serogroup, whose complete OPS and the core of LPS has been chemically characterized (Pieretti et al., 2010), and the whole-genome sequence of *P. shigelloides* 302-73 have been obtained in Pique et al. (2013). *P. shigelloides* O17 is one of the most frequently encountered serovar, which is involved with protection against shigellosis as it carries a LPS in the cell walls that is also common to *S. sonnei* (Sayeed et al., 1992).

In this study, 12 O-antigen gene clusters of *P. shigelloides*, serogroups O2, O10, O12, O23, O25, O26, O32, O33, O34, O66, O75, and O76, were sequenced and analyzed. The genes that control O-antigen synthesis are present as chromosomal gene clusters that maps between *rep* and *aqpZ*, and most of the synthesis and translocation of OPS occurs within the Wzx/Wzy pathway, with the exception of O12, O25, and O66, which use ABC transporter pathway. Phylogenetic analysis of *wzx* and *wzy* show that the *wzx* and *wzy* genes are specific to individual O-antigens and can be used as targets in molecular typing.

In summary, the current study: (1) has determined 12 O-antigen gene clusters of *P. shigelloides* serogroups O2, O10, O12, O23, O25, O26, O32, O33, O34, O66, O75, and O76; (2) has analyzed the evolutionary relationship of the O-antigen gene clusters in combination with the published O-antigen-specific gene data of *P. shigelloides* O1; (3) has established a rapid, bead-based suspension array detection technique using the *wzx, wzy*, or *wzm* genes of 12 different serogroups of *P. shigelloides* as the molecular serotyping markers. The suspension array method has been tested for its specificity and sensitivity and found to be suitable for applications in the fields of clinical research, diagnosis, treatment, epidemiology, and prevention of diseases.

## Materials and Methods

### Bacterial Strains

The 12 *P. shigelloides* O-standard reference strains employed in this study are listed in Table 1.

**Table 1:** Bacterial strains used in this study.

Bacterium	Lab number	Original number	Original source	Total number
*Plesiomonas shigelloides* O2H1a1c	G5877	CNCTC Aer 33/89	CNCTC	1
*Plesiomonas shigelloides* O10h41	G5892	CNCTC Aer 58/89	CNCTC	1
*Plesiomonas shigelloides* O12h35	G5890	CNCTC Aer 43/89	CNCTC	1
*Plesiomonas shigelloides* O23H1a1c	G5263	A1	Italy	1
*Plesiomonas shigelloides* O25H3	G5879	CNCTC Aer 37/89	CNCTC	1
*Plesiomonas shigelloides* O26H1a1c	G5889	CNCTC Aer 36/89	CNCTC	1
*Plesiomonas shigelloides* O32H37	G5880	CNCTC Aer 61/89	CNCTC	1
*Plesiomonas shigelloides* O33H38	G5881	CNCTC Aer 62/89	CNCTC	1
*Plesiomonas shigelloides* O34H34	G5882	CNCTC Aer 35/89	CNCTC	1
*Plesiomonas shigelloides* O66H3	G5270	H3	Italy	1
*Plesiomonas shigelloides* O75H34	G5885	CNCTC Aer 3/65	CNCTC	1
*Plesiomonas shigelloides* O76H39	G5886	CNCTC Aer 55/89	CNCTC	1

*CNCTC, Czechia National Collection of Type Cultures, the Czechia. Department of Environmental Sciences, Naples, Italy.*

### Genomic DNA Extraction

All *P. shigelloides* strains were cultured in Tryptone Soya Broth (TSB) (QingDao ShuiRi Bio-Technologies Co., Ltd., Qingdao, China) at 37°C overnight with shaking. Genomic DNA was prepared by Bacteria Gen DNA Kit (CWBiotech, Beijing, China).

### Library Preparation for Illumina Sequencing

A library for Illumina Paired-End sequencing was prepared from 5 μg DNA using a Paired-End DNA Sample Prep Kit (Pe-102-1001, Illumina Inc., Cambridge, United Kingdom). DNA was fragmented by nebulization for 6 min at a pressure of 32 psi. For end-repair and phosphorylation, sheared DNA was purified using the QIAquick Nucleotide Removal Kit (Qiagen, Crawley, United Kingdom). The end-repaired DNA was A-tailed and adaptors were ligated according to the manufacturer’s instructions.

### Draft Genome Sequencing, Assembling, and Annotation

As reported previously (Hu et al., 2013), the whole-genome sequencing was performed using an Illumina/Solexa genome analyzer IIx (Illumina, San Diego, CA, United States). *De novo* assembly was performed using SOAP *de novo* (Xie et al., 2014). Gene prediction and annotation were carried out using the NCBI Prokaryotic Genome Annotation Pipeline (Tatusova et al., 2016). The Staden package and the Artemis program were used for sequence assembly and gene annotation, respectively. The BlockMaker program was used to search for conserved motifs. BLAST and PSI-BLAST (Altschul et al., 1997) were used to search sequence databases, including the GenBank database and the Pfam protein motif database (Bateman et al., 2002), to identify potential gene functions. The program TMHMM 2.0 (Krogh et al., 2001) was used to identify potential transmembrane segments. Sequence alignments and comparisons were performed using the ClustalW program (Thompson et al., 1994). For sugar pathway genes, a BLAST search against the UniProt/SwissProt database was used to confirm the allocation of the genes by pathway.

### Phylogenetic Analysis

A phylogenetic tree was constructed using the neighbor-joining method and plotted by the Molecular Evolutionary Genetics Analysis (MEGA) 6.0 software package. Bootstrap analysis was carried out based on 1,000 replicates.

### Target Genes and Primer Design

The target genes for primer and probe design were *wzx* genes of *P. shigelloides* O2, O10, and O26, *wzy* genes of *P. shigelloides* O23, O32, O33, O34, O75, and O76, and *wzm* genes of *P. shigelloides* O12, O25, and O66 (Table 2). Primers were designed by Primer Premier (Yang et al., 2018). The forward primer was biotinylated at the 5′-end to allow binding to the reporter dye streptavidin-R-phycoerythrin for detection on a Bio-Plex platform. All the primer sequences used for the multiplex PCR are listed in Table 2.

**Table 2:** Primers and probes.

Group	Target species	Target gene	Primer/ probe name	Sequence (5′-3′)	Length (nt)	Tm (C)	PCR product size (bp)
A	O12	*wzm*	wl71881	AAAGGAAGGCTATGCGTTC	21	55.2	184
			wl71882	GAAAGGTTCATTAGTCTGGGA	19	54.7	
			OA-5521	ACTCCACCATCCAAGAGAAAT	21	53.7	
	O25	*wzm*	wl71869	AGAGACTTTCCTCAAATGGTAA	22	53.1	172
			wl71870	TACACCTCTGGAACTTTACCT	21	51.0	
			OA-5515	AGAGCCCCGCTGATAGG	17	55.4	
	O26	*wzx*	wl71879	AAATGTCACCATCTTTACGG	20	52.6	351
			wl71880	GCACTCTGACCTGCCTGATA	20	55.7	
			OA-5520	GTATACCAGCGCGAGTACATTT	22	57.0	
	O33	*wzy*	wl71871	TAAGCAGAAACCGTCTAACC	20	52.5	256
			wl71872	CAGCTCAGTAGGTACGAGAAAT	22	53.7	
			OA-5516	CAGAAACCGTCTAACCACTACAT	23	55.9	
	O75	*wzy*	wl71875	TAACTCTTTTCTATGTGCCGA	21	53.2	82
			wl71876	GGCATTACACAGAGCAAGAT	20	52.5	
			OA-5518	ATGATCGGCCATCGTCAT	18	56.0	
	O76	*wzy*	wl71877	CACATATGGAAAAAACTGGG	20	53.1	205
			wl71878	AAGAAACAGCCGACCAGATA	20	54.9	
			OA-5519	GGCGCACGTATCGAAGG	17	57.8	
B	O2	*wzx*	wl72935	AATGGATTTGTGCTTTTTCTT	21	54.0	263
			wl72936	GCAAGTTGCCACTTAGTGTTA	21	53.5	
			OA-5851	CAGGCCCATTGAACCAGT	18	55.8	
	O10	*wzx*	wl72937	AATAATAGTGGCTGGTTAATGTT	23	53.2	231
			wl72938	AAATGCCGTTCCGAGTAT	18	52.7	
			OA-5852	AACCAAAGCGGGTGAGATA	19	55.4	
	O23	*wzy*	wl72931	CTACTGAATCCAACCACGC	19	53.4	192
			wl72932	ATTTTGTGGATTATACATTGGA	22	52.2	
			OA-5849	CACCAAGCCAAGTCGAGAA	19	56.6	
	O32	*wzy*	wl72925	AGTCAATAACATACCCAAACG	21	52.3	290
			wl72926	AGAGAGCCCTTCTATTCCAA	20	53.4	
			OA-5846	GTCGCAGACTGTTGACTAAGAA	22	54.9	
	O34	*wzy*	wl72927	GGTCTCCGCAAAAGATAGTC	20	54.0	317
			wl72928	TTTATCGCTTACTCTTGCTCA	21	53.6	
			OA-5847	TTAACGCCAACCAGGTAGTAG	21	55.3	
	O66	*wzm*	wl72933	CACATTCGTGTTTAGTGTGGT	21	53.2	214
			wl72934	TCCAAAACTTGAGAACACCA	20	53.5	
			OA-5850	AGGCAGCGGTTCAAAGC	17	56.6	

### Multiplex PCR and Labeling of the Target Genes

Multiplex PCR was carried out in two groups of six serogroups each; Group A consists of *P. shigelloides* O12, O25, O26, O33, O75, and O76; Group B consists of *P. shigelloides* O2, O10, O23, O32, O34, and O66. The multiplex PCR amplification was performed with 50 μL of a reaction mixture that consisted of 100 ng of DNA, 25 μL of HotStar Taq Master Mix (Qiagen), 0.25 μL of the forward primer, and 1.0 μL of the reverse one, with an exception of O25 with double the amount of both primers. Reaction parameters were: 95°C for 5 min; 35 cycles of 94°C for 30 s, 54°C for 45 s, and 72°C for 50 s; and a final extension at 72°C for 5 min. An aliquot of 2 μL of PCR product was run on an agarose gel to examine the amplified DNA. After amplification, the samples were stored at −20°C until used.

### Probe Design and Bead Coupling

All species-specific probes were based on the sequence data from the lab data. Multiple-sequence alignments were carried out using BioEdit version 7.0 software. These species-specific probes were newly designed (Table 2). Probes were synthesized with a 5′-end amino C-12 modification (AuGCT, Beijing, China) and coupled to carboxylated beads (Bio-Rad Laboratories, Hercules, CA, United States) as described by the manufacture’s manual. Briefly, a total of 2.5 × 10^5^ carboxylated beads were suspended in 8.5 μL of 0.1 M MES (pH 4.5) with 2 μL of 0.1 nmol/μL oligonucleotide probes. A 2.5 μL of 10 mg/mL freshly prepared EDC [1-ethyl-3-(3-dimethylaminopropyl) carbodiimide] was added, and vortexed immediately, then incubated at room temperature in the dark for 30 min. After being washed by 0.02% Tween 20 and 0.1% SDS, pellets were centrifuged and resuspended in 20 μL of TE (pH 8.0), and stored at 4°C in the dark until used.

### Hybridization and Staining

A bead mix set was prepared, consisting of 2,500 beads for each of the 10 probes in 1.5× tetramethylammonium chloride (TMAC) solution (Sigma, St. Louis, MO, United States) that contained 4.5 M TMAC, 0.15% Sarkosyl, 75 mM Tris–HCl (pH 8.0), and 6 mM EDTA (pH 8.0) solution. A total of 17 μL of biotinylated amplicon was added to 33 μl of the bead mix. Amplicon and bead mixture were denatured at 95°C for 5 min and allowed to hybridize at 57°C for 15 min. The mixture was centrifuged at 8,000 rpm and the supernatant carefully discarded, then the beads were resuspended in 75 μL of 1× TMAC solution containing 10 ng/mL streptavidin-R-phycoerythrin (Molecular Probes, Eugene, OR, United States) and incubated for 10 min at 57°C.

### Bead Analysis

The fluorescence intensity of the beads was analyzed in a Bio-Plex 100 suspension array system (Bio-Rad Laboratories). The median fluorescence intensities (MFI) were calculated from 100 replicate measurements with a digital signal processor and BioPlex Manager 4.1 software. A positive signal was defined as MFI is at least >500, and the signal/background ratio (S/B ratio = MFI/Blank) is greater than 3.0.

### Sensitivity of the Multiplex PCR Method

A serial ten-fold dilution of the genomic DNA of *P. shigelloides* O26 and O75, ranging from 1.0 fg/uL, 10 fg/uL, 100 fg/uL, 1.0 pg/uL, 10 pg/uL, 100 pg/uL, 1.0 ng/uL, 10 ng/uL, and 100 ng/uL, were used as the template to test the sensitivity of the suspension array assay.

### Nucleotide Sequence Accession Number

The DNA sequences of the O-antigen gene clusters of 12 O-antigen gene clusters of *P. shigelloides* serogroups O2, O10, O12, O23, O25, O26, O32, O33, O34, O66, O75, and O76 have been deposited in GenBank under the accession numbers MK551179-MK551190, respectively.

## Results

### Allocation and Extraction of OPS Clusters From *P. shigelloides* Genome Sequences

**FIGURE 1:**
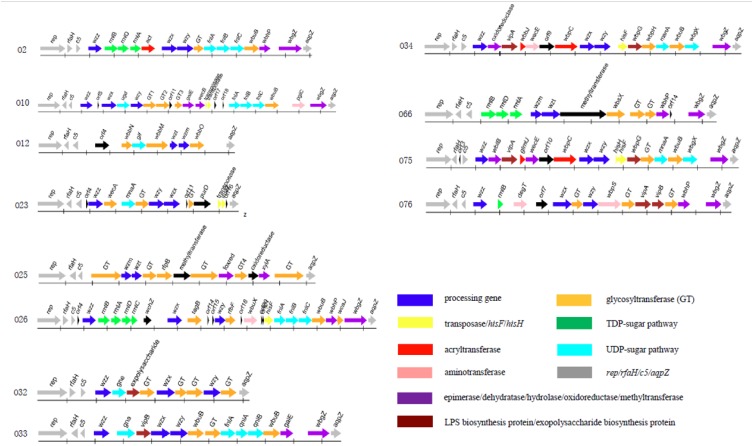
The O-antigen gene clusters of 12 *P. shigelloides* serogroups O2, O10, O12, O23, O25, O26, O32, O33, O34, O66, O75, and O76.

**FIGURE 2:**
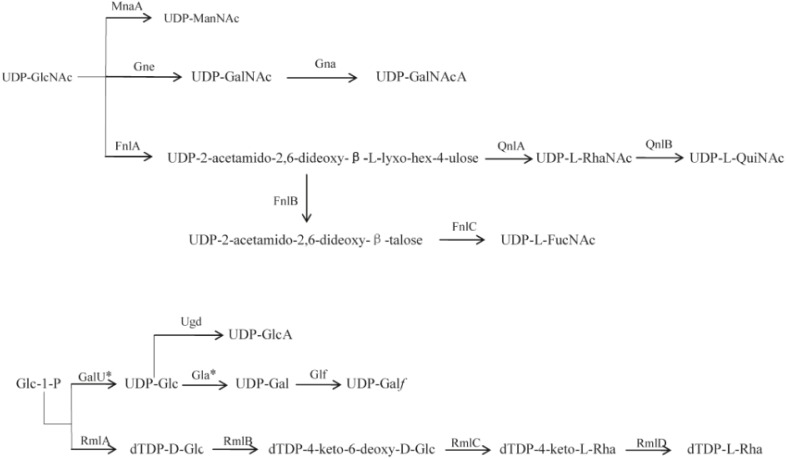
Proposed biosynthesis pathways for sugars in the 12 *P. shigelloides* serogroups clusters. Putative pathways are denoted by a broken line. MnaA, UDP-N-acetylglucosamine-2-epimerase; Gne, UDP-N-acetylglucosamine-4-epimerase; Gna, UDP-GalNAcA synthetase; FnlA, 4,6-dehydratase, 3- and 5-epimerase; FnlB, reductase; FnlC, C-2 epimerase; QnlA, dTDP-4-dehydrorhamnose reductase; QnlB, C-2 epimerase; GalU^∗^, UTP-glucose-1-phosphate uridylyltransferase; Ugd, UDP-glucose 6-dehydrogenase; Gla^∗^, UDP-galacturonase; Glf, UDP-galactopyranose mutase; RmlA, glucose-1-phosphate thymidylyltransferase; RmlB, dTDP-D-glucose 4,6-dehydratase; RmlC, dTDP-4-keto-6-deoxy-D-glucose 3,5-epimerase; RmlD, dTDP-6-deoxy-L-mannose-dehydrogenase. D-GlcNAc, 2-acetamido-2-deoxy-D-glucose; D-ManNAc, 2-acetamido-2-deoxy-D-mannose; D-GalNAc, 2-acetamido-2-deoxy-D-galactose; D-GalNAcA, 2-Acetamido-2-deoxy-D-galacturonic acid; L-RhaNAc, 2-acetamido-2,6-dideoxy-L-mannose (N-acetyl-L-rhamnosamine); L-QuiNAc, 2-Acetamido-2-deoxy-L-quinovose (2-acetamido-2,6-dideoxy-L-glucose); L-FucNAc, 2-Acetamido-2-deoxy-L-fucose; D-GlcA, D-glucuronic acid; D-Glc, D-glucose; D-Gla^∗^, D-galactose; D-Gal*f*, D-galactofuranose; L-Rha, L-rhamnose (6-deoxy-L-mannose). ^∗^indicates the genes that were found outside the cluster.

The O-antigen gene cluster of 12 *P. shigelloides* serogroups were obtained and extracted from the type strains of *P. shigelloides* serogroups O2, O10, O12, O23, O25, O26, O32, O33, O34, O66, O75, and O76 (Table 1). The clusters located between *rep* and *aqpZ* at the chromosome of these 12 isolates had 22,165, 27,407, 16,526, 15,506, 20,682, 29,124, 14,430, 21,362, 25,286, 21,030, 25,342, and 24,898 bp, respectively. Characteristics of the 18, 25, 11, 17, 16, 30, 13, 17, 20, 16, 22, and 18 open reading frames (orfs) of these 12 isolates were identified to each of the serogroups (Figure 1 and Supplementary Tables S1–S12). The functions were assigned based on their similarities to genes of known functions associated with sugar synthesis, sugar transfer, O-antigen processing, and others from available databases (Figure 1, 2).

### O-Antigen Cluster of *P. shigelloides* O2

For the synthesis of UDP-L-FucNAc, FnlABC is reportedly involved in synthesizing O-polysaccharides in several species, e.g., in *Salmonella* O47, O48, and O61 (Liu et al., 2014). The set of orf12, orf13, and orf14 in the cluster of *P. shigelloides* O2 shares 93% identity with FnlA of *P. shigelloides* WP_010862887, 65 and 84% with FnlBC of *E. coli*, indicating the existence of a rare sugar, UDP-L-FucNAc. Rhamnose is widely present as a bacterial surface polysaccharide, and its biosynthesis pathway is well known to require four enzymes that are encoded by *rmlABCD* in the polysaccharide gene clusters of *E. coli*, *Shigella*, and *Salmonella* (Liu et al., 2008, 2014). For *P. shigelloides* O2, the set of orf5, orf6, and orf7 of *P. shigelloides* O2 shows around 90% identity to RmlBDA in *E. coli*, indicating the existence of another rare sugar, dTDP-L-Rha. The orf9 shows 39% identity to Wzx in *Escherichia coli* and the orf10 shows 40% identity to Wzy in *Pseudomonas mucidolens*. The set of *wzx* and *wzy* genes suggests the presence of Wzx/Wzy pathway related O-antigen processing. There are two GTs encoded by the orf11 and orf15, which share 37% identity with GT family 2 protein of *Pseudomonas* sp. NFR02, and 58% identity with GT/WbuB of *Burkholderia territorii*.

### O-Antigen Cluster of *P. shigelloides* O10

The set of FnlABC in the cluster of *P. shigelloides* O10 shares 98% identity with FnlA of *P. shigelloides*, 63% identity with FnlB of *E. coli*, and 99% identity with FnlC of *P. shigelloides*, indicating the existence of the rare sugar, UDP-L-FucNAc. The function of Ugd is to convert UDP-Glc to UDP-GlcA. The orf7 shares more than 94% identity with Ugd in *Shewanella algae*. The orf6 shows 79% identity to Wzx in *Shewanella decolorationis* and the orf8 shows 32% identity to Wzy in *Marinobacter* sp. EN3. The set of *wzx* and *wzy* genes suggests the presence of Wzx/Wzy pathway related O-antigen processing. There are three GTs encoded by the orf9, orf10, and orf12 which share 40% identity with GT family 2 protein of *Prosthecochloris* sp. CIB 2401, 35% identity with GT family 1 protein of *P. shigelloides*, and 88% identity with GT of *S. algae*, respectively.

### O-Antigen Cluster of *P. shigelloides* O12

There is no rare sugar in the cluster of *P. shigelloides* O12. The function of Glf is to convert UDP-Gal to UDP-Gal*f*. The orf6 in the cluster shares 79% identity with the corresponding gene of *Klebsiella pneumoniae*. The set of *wzm* and *wzt* genes suggests the presence of ABC transporter pathway related O-antigen processing. There are three GTs encoded by the orf5, orf7, and orf10 which share 50% identity with GT/WbbN of *Serratia liquefaciens*, 67% identity with GT/WbbM of *K. pneumoniae* subsp. *rhinoscleromatis* SB3432, and 57% identity with GT/WbbO of *Serratia* sp. Leaf50, respectively.

### O-Antigen Cluster of *P. shigelloides* O23

The function of MnaA is to convert UDP-GlcNAc to UDP-ManNAc. MnaA in *P. shigelloides* O23 share more than 81% identity with the one in *E. coli*, indicating there is a rare sugar UDP-ManNAc in the structure. The orf10 shows 27% identity to Wzx in *E. coli* S88 and the orf9 shows 29% identity to Wzy in *Salmonella enterica*. The set of *wzx* and *wzy* genes suggests the presence of Wzx/Wzy pathway related O-antigen processing. WecA in *P. shigelloides* O23 shares more than 93% identity with the corresponding gene of *P. shigelloides*. There is one GT encoded by the orf8 which shares 42% identity with GT family 4 protein of *Vibrio cholerae*. There are two transposes encoded by the orf14 and orf15. As Duan et al. (2016) reported in most *E. coli* and *Shigella* strains, the first sugar residue in the O-unit is GlcNAc or GalNAc, and the IT (Initial transferase) encoded by *wecA*, which is responsible for initiating the synthesis of GlcNAc- and GalNAc- initiated OPS. Usually, the *wecA* gene is located outside the cluster and acts as a UDP-GlcNAc:undecaprenyl phosphate GlcNAc-1-phosphate transferase, however, it presents in the O23 gene cluster.

### O-Antigen Cluster of *P. shigelloides* O25

There is no rare sugar in the cluster of *P. shigelloides* O25. The set of *wzm* and *wzt* genes suggests the presence of the ABC transporter pathway related O-antigen processing. Interestingly, there are six GTs encoded by the orf4, orf7 orf8, orf10, orf12, and orf15 which share 54% identity with GT family 1 protein of *Enterobacteriaceae*, 56% identity with GT family 1 protein of *Citrobacter europaeus*, 67% identity with GT/RfbP of *C. europaeus*, 67% identity with GT family 1 protein of *C. europaeus*, 65% identity with GT family 2 protein of *Serratia* sp. DD3, and 77% identity with GT family 2 protein of *Duganella* sp. Root336D2, respectively.

### O-Antigen Cluster of *P. shigelloides* O26

The orf6 and orf7 of *P. shigelloides* O26 share 89 and 95% identity with RmlB and RmlA of *V. cholerae*, and the orf8 and orf9 share 85 and 83% identity with RmlD and RmlC of *Vibrio anguillarum*, indicating the presence of a rare sugar, dTDP-L-Rha. The set of orf23, orf24, and orf25 in the cluster shares 93% identity with FnlA of *P. shigelloides* WP_010862887, 65 and 84% with FnlBC of *E. coli*, indicating there is a rare sugar UDP-L-FucNAc. The orf12 shows 36% identity to Wzx in *E. coli* and the orf16 shows 36% identity to Wzy in *Flavobacterium* sp. GSP27. The presence of *wzx* and *wzy* genes suggests the presence of Wzx/Wzy pathway related O-antigen processing in O26. Except the two GTs of orf13 and orf28 encoding TagD for teichoic acid biosynthesis and WcaJ for colanic acid biosynthesis. There are other two GTs encoded by the *orf17* and *orf26* which share 38% identity with GT/RfbF of *Shigella flexneri*, and 89% identity with GT/WbuB of *V. cholerae*, respectively.

### O-Antigen Cluster of *P. shigelloides* O32

There is no rare sugar in the cluster of *P. shigelloides* O32. The orf8 shows 45% identity to Wzx in *E. coli* and the orf11 shows 33% identity to Wzy in *S. enterica*. The set of *wzx, wzy*, and *wzz* genes suggests the presence of Wzx/Wzy pathway related O-antigen processing. The orf5 in the cluster of *P. shigelloides* O32 shares 64% identity with Gne of *Yersinia pseudotuberculosis*, indicating there is a sugar UDP- GalNAc. There are four GTs encoded by the orf7, orf9, orf10, and orf12, which share 57% identity with GT of *S. enterica*, 57% identity with GT family 2 protein of *Proteus mirabilis*, 58% identity with GT family 4 protein of *Edwardsiella tarda*, and 55% identity with GT of *Shewanella* sp. SACH, respectively.

### O-Antigen Cluster of *P. shigelloides* O33

The orf5 in the cluster of *P. shigelloides* O33 shares 83% identity with Gna of *P. shigelloides*, indicating the presence of a sugar, UDP- GalNAcA. The set of orf11, orf12, and orf13 in the cluster of *P. shigelloides* O33 shares more than 93, 69, and 88% identity with FnlA, QnlA, and QnlB of *E. coli*, indicating there is a rare sugar, UDP-L-QuiNAc. The orf7 shows 38% identity to Wzx in *E. coli* and the orf8 shows 46% identity to Wzy in *Moritella viscosa*. There are *wzx* and *wzy* genes in the cluster, indicating the presence of Wzx/Wzy pathway related O-antigen processing. There are three GTs encoded by orf9, orf10, and orf14 which share 23% identity with GT/WbuB of *Pseudomonas fluorescens*, 76% identity with GT family 1 protein of *Aeromonas fluvialis*, and 74% identity with GT/WbuB of *Shewanella xiamenensis*, respectively.

### O-Antigen Cluster of *P. shigelloides* O34

The orf16 in the cluster of *P. shigelloides* O34 shares more than 89% identity with MnaA of *V. cholerae*, indicating the presence of UDP-ManNAc. The orf11 shows 36% identity to Wzx in *Janthinobacterium* sp. CG23_2 and the orf12 shows 35% identity to Wzy in *Pseudomonas* sp. BAY1663. The set of *wzx, wzy*, and *wzz* genes suggest the presence of Wzx/Wzy pathway related O-antigen processing. There are two GTs encoded by the orf15 and orf17 which share 57% identity with GT/WbpH of *Pseudomonas* sp. CFII68, and 55% identity with GT of *Photorhabdus luminescens*.

### O-Antigen Cluster of *P. shigelloides* O66

The set of orf4, orf5 and orf6 in the cluster of *P. shigelloides* O66 share 91, 94, and 98% identity with RmlBDA of *Aeromonas* sp. indicating there is a rare sugar dTDP-L-Rha. The *galE* is the gene that is responsible for the synthesis of UDP-Gal in the gene cluster. The set of *wzm* and *wzt* genes suggest the presence of the ABC transporter pathway related O-antigen processing. There are three GTs encoded by the orf10, orf11, and orf12 which share 63% identity with GT/WbsX of *Nitrosomonas europae*, 81% identity with GT of *Aeromonas hydrophila*, and 75% identity with GT family 2 protein of *Shigella boydii*, respectively.

### O-Antigen Cluster of *P. shigelloides* O75

The MnaA in the cluster of *P. shigelloides* O75 shares more than 89% identity with the one of *V. cholerae*, indicating there is a rare sugar, UDP-ManNAc, in the structure. The orf12 shows 42% identity to Wzx and the orf13 shows 34% identity to Wzy in *Pseudomonas* sp. BAY1663. The set of *wzx, wzy*, and *wzz* genes suggests the presence of Wzx/Wzy pathway related O-antigen processing. There are two GTs encoded by the orf17 and orf19, which share 87% identity with GT of *Vibrio ordalii*, and 55% identity of GT/WbuB of *P. luminescens*.

### O-Antigen Cluster of *P. shigelloides* O76

There is no rare sugar in the cluster of *P. shigelloides* O76. The orf8 shows 51% identity to Wzx in *Acinetobacter baumannii* and the orf10 shows 45% identity to Wzy in *E. coli*. The set of *wzx, wzy*, and *wzz* genes suggests the presence of Wzx/Wzy pathway related O-antigen processing. There are three GTs encoded by the orf9, orf12, and orf15, which share 81% identity with GT family 2 protein of *Pseudomonas oleovorans*, 91% identity with GT of *V. cholerae*, and 91% identity with GT/WbuB of *V. cholerae*, respectively.

### Selection of Target Genes

**FIGURE 3:**
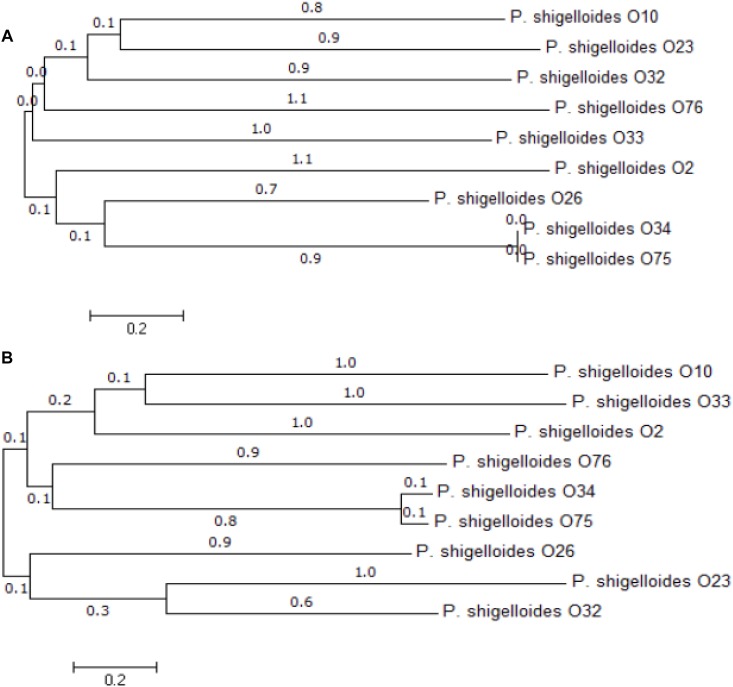
Phylogenetic analysis. An unrooted phylogenetic tree constructed by the neighbor joining method based on the *wzx* **(A)** and *wzy* **(B)** genes is shown. Bootstrap values were based on 1000 replications and only values greater than 50% are shown.

Previous studies suggest that O-antigen processing genes, *wzx* and *wzy* for Wzx/Wzy pathway or *wzm* and *wzt* for ABC transporter pathway, are highly specific to individual O-antigens (Greenfield and Whitfield, 2012; Islam and Lam, 2014). We therefore selected them as targets for PCR-based typing methods. Out of the 12 *P. shigelloides* O-antigens, three of them contain both *wzm* and *wzt*, the remaining nine contain both *wzx* and *wzy*. We have constructed the maximum likelihood phylogenetic trees using the *wzx* and *wzy* genes, and the results show the high levels of diversity of these two genes among different strains (Figure 3). The diversity of the *wzx* and *wz*y genes provided us a basis to develop molecular techniques to detect and identify different *P. shigelloides* O serogroups. Accordingly, the specific primer pairs were designed based on the *wzx* genes of O2, O10, and O26; the *wzy* genes of O23, O32, O33, O34, O75, and O76; and the *wzm* genes of O12, O25, and O66.

### Amplification and Hybridization

**FIGURE 4:**
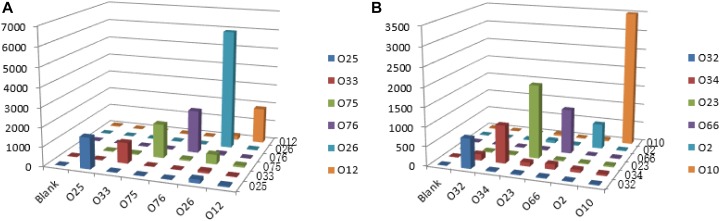
Specific detection by the xMAP bead-based multiplex assay on the 12 *P. shigelloides* O-antigens. MFI, median fluorescent intensity; the *X*-axis represents *P. shigelloides* strains of the 12 O-antigens. **(A)** Group A; **(B)** Group B.

Multiplex PCR was used to amplify and generate the single strands of individual amplicons. Under our optimized conditions, the amplicons of the 12 O-antigens were amplified simultaneously with the PCR products ranging from 82 to 351 bp in length for clear hybridization signals. For all of the 12 strains tested, the S/B ratio for each probe tested against its homologous DNA was significantly greater than the ratio that was obtained when the probes tested against non-homologous DNA. The S/B ratios of the positive samples are set as greater than 3.0. No cross reactivity was observed for any probe that was tested against non-homologous DNA (Figure 4).

### Sensitivity of Detection With Genomic DNA

**FIGURE 5:**
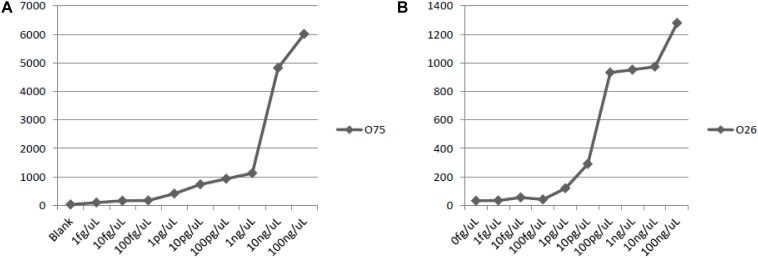
Sensitivity of detection with genomic DNA of O75 and O26. **(A)** sensitivity of O75 genomic DNA; **(B)** sensitivity of O26 genomic DNA.

The lowest amount of DNA that produces positive signals were: 100 pg DNA for *P. shigelloides* O26, and 10 pg DNA for *P. shigelloides* O75 (Figure 5). And the sensitivity of the assay with genomic DNA was set at 1.0 ng of DNA.

## Discussion

L-Rha is widely distributed in O-antigens of bacteria, and the four *rml* genes related to the biosynthesis are usually grouped together and easily identified in a range of species. In *E. coli* and *Salmonella*, the genes are generally in the order of *rmlBDAC* at the 5′ end of the O-antigen gene cluster downstream of *wzz* (Liu et al., 2014). Out of the 12 *P. shigelloides* O-antigens studied in this report, L-Rha is present in three of them, O2, O26, and O66. *rmlC* is present in O26 cluster, and the set of four *rml* genes is in the order of *rmlBADC* at the 5′ end of the O-antigen gene cluster downstream of *wzz*, *rmlC* is absent in O2 and O66 clusters, and the rest of the three genes of the two O-antigens are in the order of *rmlBDA* at the 5′ end of the O-antigen gene cluster downstream of *wzz*. The two missing *rmlC* of O2 and O66 outside the O-antigen clusters are likely located somewhere in the chromosome. It is in line with the fact that *rmlB*, *rmlD*, and *rmlA* genes of *E. coli* are conserved and have characteristics of housekeeping genes (Li and Reeves, 2000), while the *rmlC* gene is more varied and O-antigen specific.

The O-antigen gene cluster of strain 302-73 (serotype O1) and the chemical structure of the O-specific chains have been reported (Aquilini et al., 2013; Pique et al., 2013) with the genes *rep* and *aqpZ* located at the two ends of the O-antigen cluster. The *fnlABC*, *pglB*, and *pglD* in the cluster indicate the presence of UDP-L-FucNAc, PneNAc, and QuiNAcHb in the structure. And SDS-PAGE showed that both *pglD* and *fnlA* mutants lack the O-antigen LPS by their gel profile (Aquilini et al., 2013). The O-antigen gene cluster and chemical structure of O-specific chain of PCM2231 (serotype O17) have also been studied (Chida et al., 2000; Maciejewska et al., 2013). The genes *rep* and *aqpZ* were found in the O-antigen cluster anchors. The presence of *wbgVX* and *wbgZ* indicate there is a UDP-D-FucNAc4N and UDP-L-AltNAcA. All of the 12 *P. shigelloides* strains used in this report are reference strains for their corresponding serogroups, and are in line with the O1 and O17. Out of these 12 O-antigen clusters, several rare sugars can be deduced based on the highly homologous sugar synthetic genes, for example, dTDP-L-Rha in O2, O26, and O66; UDP-L-FucNAc in O2, O10, and O26; UDP-L-QuiNAc in O33; and UDP-ManNAc in O23, O34, and O75. In addition, there are two rare sugars of dTDP-L-Rha and UDP-L-FucNAc in both O2 and O26. However, the existence of these rare sugars need to be confirmed by chemical structure determination, such as, NMR spectrum (Perepelov et al., 2016).

In most *E. coli* and *Shigella* strains WecA, which is encoded by a gene in the enterobacterial common antigen (ECA) gene cluster, is responsible for initiating the synthesis of GlcNAc- and GalNAc-initiated O-antigens by transferring GlcNAc-1-phosphate to the UndP carrier (Liu et al., 2008). The existence of *wecA* in O23 cluster indicating the existence of either GlcNAc or GalNAc in its chemical structure. We used O23 WecA to blast the genomes of the other 11 *P. shigelloides* and found that all the other 11 *wecA*s are located in the chromosomes outside the O-antigen gene clusters with similarity of 37% and *e*-value of 1.00E-66.

In the 12 *P. shigelloides* serogroups O-antigens, ABC transporter pathways encoded by *wzm/wzt* are employed for O-antigen processing of O12, O25, and O66; and Wzx/Wzy pathways encoded by *wzx/wzy* are employed for O-antigen processing of the other nine ones, O2, O10, O23, O26, O32, O33, O34, O75, and O76.

We took efforts to improve the reproducibility and sensitivity of the suspension array. We have compared three methods of amplification, (1) in the two-step PCR method, at the first step, the same concentration of forward and reverse primers were used to amplify the target genes at the first step; followed by the labeling of single–strand DNA using the reverse primers only; (2) the one-step symmetry PCR method, which uses the same concentration of both forward and reverse primers for PCR reactions; and (3) the one-step asymmetry PCR method, the different concentration of forward and reverse primers were used for PCR reactions. We found the one-step asymmetry PCR method achieved the best results. Also, in order to obtain a better hybridization signal, the PCR amplification fragments were kept shorter than 350 bp. The sensitivity of the method reaches 100 pg DNA for *P. shigelloides* O26, and 10 ng DNA for *P. shigelloides* O75.

Shepherd et al. (2000) reported that *S. sonnei* gained its current plasmid-borne O-antigen genes from *P. shigelloides* O17 in a recent event. As described in this report, we established the method to distinguish them by targeting *wzy* and *wbgV*. We expected that the suspension array is able to detect and dissect new serogroups of recombinant *P. shigelloides* that may arise from time to time.

However, the array has its limitations as the design probes requires prior knowledge of the sequence. In summary, the results in the study demonstrated that the probes and primers worked well on target strains and generated the specific signals and the suspension array is able to provide an easy, rapid, and accurate detection for *P. shigelloides* to facilitate better monitor the disease.

## Author Contributions

BC conceived the project. DX and XG purchased the strains. DX prepared the sample DNA, performed the sequence analyses, and developed the molecular serotyping system. XW and QL conducted the bioinformatics analyses. DX, KN, and FJ developed the multiplexed Luminex – based array. BC and DX prepared the manuscript. All authors read and approved the final manuscript.

## Conflict of Interest Statement

The authors declare that the research was conducted in the absence of any commercial or financial relationships that could be construed as a potential conflict of interest.
