# Supplementary material for: O-Antigen Gene Clusters of Plesiomonas shigelloides Serogroups and Its Application in Development of a Molecular Serotyping Scheme
Source: Front Microbiol. 2019 Apr 10;10:741. doi: 10.3389/fmicb.2019.00741 (PMC6467956; doi:10.3389/fmicb.2019.00741)
Supplement: Supplementary file 1 [file Data_Sheet_1.docx]

**Supplementary material**

**Journal name: Frontiers in Microbiology**

**Article title:**

**O-antigen gene clusters of** ***Plesiomonas shigelloides* serogroups and its application in development of a molecular serotyping scheme**

**Author names:**

**Daoyi Xi^1,2,3,4†^, Xiaochen Wang^1,2,3,4†^, Kexin Ning^1,2,3,4^, Qian Liu^1,2,3,4^, Fuyi Jing^1,2,3,4^, Xi Guo^1,2,3,4^ and Boyang Cao^1,2,3,4^***

**Affiliations:**

1. **Key Laboratory of Molecular Microbiology and Technology of the Ministry of Education, TEDA College, Nankai University, Tianjin, P. R. China**
2. **TEDA Institue of Biological Sciences and Biotechnology, Nankai University, Tianjin, P. R. China**
3. **Tianjin Research Center for Functional Genomics and Biochips, TEDA College, Nankai University, Tianjin, P. R. China**
4. **Tianjin Key Laboratory of Microbial Functional Genomics, TEDA College, Nankai University, Tianjin, P. R. China**

**^†^ These authors have contributed equally to this work.**

***Corresponding Author: Tel: 86-22-66229583, E-mail address:** [**boyangcao@nankai.edu.cn**](mailto:wanglei@nankai.edu.cn)

| **Table S1** Characteristics of the ORFs of *P. shigelloides* O2H1a1c O antigen gene cluster | | | | | | |
| --- | --- | --- | --- | --- | --- | --- |
| **ORF#** | **Gene name /location /direction** | **No. of amino acids** | **G+C content(%)** | **Similar protein/strain/GenBank accession No.** | **% Identity/% Similarity (No. of aa overlap)** | |
| O2-01 | *rep* | 676 | 55.0 | DNA helicase Rep | 99/99(675) | |
|  | (1-2031) |  |  | [*Plesiomonas shigelloides*] | | |
|  | + |  |  | WP_010862901 | | |
| O2-02 | *rfaH* | 136 | 52.2 | transcription/translation regulatory transformer protein RfaH | 95/98(134) | |
|  | (2186-2596) |  |  | [*Plesiomonas shigelloides*] | | |
|  | + |  |  | [WP_064977063](https://www.ncbi.nlm.nih.gov/protein/1039817755?report=genbank&log$=protalign&blast_rank=1&RID=04TV1M5W014) | | |
| O2-03 | *c5* | 108 | 55.0 | MULTISPECIES: cytochrome c5 family protein | 96/97(105) | |
|  | (2932-3258) |  |  | [*Plesiomonas*] | | |
|  | + |  |  | [WP_081632838](https://www.ncbi.nlm.nih.gov/protein/1174959619?report=genbank&log$=protalign&blast_rank=2&RID=04TV1M5W014) | | |
| O2-04 | *wzz* | 369 | 36.9 | Wzz | 88/92(336) | |
|  | (3900-5009) |  |  | [*Plesiomonas shigelloides*] | | |
|  | + |  |  | [AAG17407](https://www.ncbi.nlm.nih.gov/protein/10442654?report=genbank&log$=protalign&blast_rank=2&RID=0UB63BGG014) | | |
| O2-05 | *rmlB* | 361 | 45.4 | dTDP-glucose 4,6-dehydratase | 89/93(336) | |
|  | (5242-6327) |  |  | [*Plesiomonas shigelloides*] | | |
|  | + |  |  | [WP_084977040](https://www.ncbi.nlm.nih.gov/protein/1183756425?report=genbank&log$=protalign&blast_rank=11&RID=04TV1M5W014) | | |
| O2-06 | *rmlD* | 295 | 59.3 | dTDP-4-dehydrorhamnose reductase | 89/95(281) | |
|  | (6327-7214) |  |  | [*Aeromonas caviae*] | | |
|  | + |  |  | [WP_010675335](https://www.ncbi.nlm.nih.gov/protein/498361179?report=genbank&log$=protalign&blast_rank=1&RID=04TV1M5W014) | | |
| O2-07 | *rmlA* | 292 | 50.5 | glucose-1-phosphate thymidylyltransferaseRfbA | 90/95(279) | |
|  | (7327-8205) |  |  | [*Aeromonas caviae*] | | |
|  | + |  |  | [WP_042017499](https://www.ncbi.nlm.nih.gov/protein/754630553?report=genbank&log$=protalign&blast_rank=9&RID=04TV1M5W014) | | |
| O2-08 | *act* | 367 | 58.0 | acyltransferase | | 35/53(185) |
|  | (8253-9356) |  |  | [*Burkholderia territorii*] | | |
|  | + |  |  | [WP_081053062](https://www.ncbi.nlm.nih.gov/protein/1173159821?report=genbank&log$=protalign&blast_rank=1&RID=04TV1M5W014) | | |
| O2-09 | *wzx* | 415 | 31.3 | Wzx | 39/60(248) | |
|  | (9911-11158) |  |  | [*Escherichia coli*] | | |
|  | + |  |  | [AFD21164](https://www.ncbi.nlm.nih.gov/protein/379976239?report=genbank&log$=protalign&blast_rank=1&RID=04TV1M5W014) | | |
| O2-10 | *wzy* | 444 | 36.7 | [oligosaccharide repeat unit polymerase](https://blast.ncbi.nlm.nih.gov/Blast.cgi#alnHdr_1180957505) | 40/59(248) | |
|  | (11158-12492) |  |  | [*Pseudomonas mucidolens*] | | |
|  | + |  |  | [WP_084378142](https://www.ncbi.nlm.nih.gov/protein/WP_084378142.1?report=genbank&log$=protalign&blast_rank=1&RID=74XAH7J4014) | | |
| O2-11 | *GT* | 273 | 31.9 | glycosyltransferase family 2 protein  [*Pseudomonas* sp. NFR02] | 37/57(154) | |
|  | + |  |  | [WP_080960467](https://www.ncbi.nlm.nih.gov/protein/1172881455?report=genbank&log$=protalign&blast_rank=1&RID=04TV1M5W014) | | |
| O2-12 | *fnlA* | 345 | 38.8 | UDP-N-acetylglucosamine 4,6-dehydratase | 93/97(335) | |
|  | (13347-14384) |  |  | [ *Vibrio cholerae* ] | | |
|  | + |  |  | [WP_010862887](https://www.ncbi.nlm.nih.gov/protein/499149577?report=genbank&log$=protalign&blast_rank=1&RID=04TV1M5W014) | | |
| O2-13 | *fnlB* | 368 | 38.1 | FnlB | 65/81(300) | |
|  | (14385-15491) |  |  | [*Escherichia coli*] | | |
|  | + |  |  | [AAV74536](https://www.ncbi.nlm.nih.gov/protein/56123299?report=genbank&log$=protalign&blast_rank=75&RID=0UB63BGG014) | | |
| O2-14 | *fnlC* | 376 | 41.2 | FnlC | 84/92(348) | |
|  | (15508-16638) |  |  | [*Escherichia coli*] | | |
|  | + |  |  | [AAV74546](https://www.ncbi.nlm.nih.gov/protein/56123310?report=genbank&log$=protalign&blast_rank=100&RID=0UB63BGG014) | | |
| O2-15 | *wbuB* | 309 | 41.9 | GlycosyltransferaseWbuB | 58/78(319) | |
|  | (16640-17869) |  |  | [ *Burkholderia territorii* ] | | |
|  | + |  |  | [WP_016960220](https://www.ncbi.nlm.nih.gov/protein/515526980?report=genbank&log$=protalign&blast_rank=1&RID=04TV1M5W014) | | |
| O2-16 | *wbhP* | 304 | 40.6 | UDP-glucose 4-epimerase | 63/78(235) | |
|  | (17866-18780) |  |  | [*Vibrio cholerae*] | | |
|  | + |  |  | [WP_046127202](https://www.ncbi.nlm.nih.gov/protein/806463929?report=genbank&log$=protalign&blast_rank=1&RID=04TV1M5W014) | | |
| O2-17 | *wbgZ* | 623 | 44.3 | WbgZ | 88/92(576) | |
|  | (19474-21345) |  |  | [*Shigella sonnei*] | | |
|  | + |  |  | [AGI19337](https://www.ncbi.nlm.nih.gov/protein/471846220?report=genbank&log$=protalign&blast_rank=5&RID=0UB63BGG014) | | |
| O2-18 | *aqpZ* | 233 | 55.3 | MULTISPECIES: aquaporin Z | 100/100(233) | |
|  | (21464-22165) |  |  | [*Plesiomonas*] | | |
|  | + |  |  | [WP_010862878](https://www.ncbi.nlm.nih.gov/protein/499149568?report=genbank&log$=protalign&blast_rank=1&RID=04TV1M5W014) | | |

| **Table S2** Characteristics of the ORFs of *P. shigelloides* O10H41 O antigen gene cluster | | | | | | |
| --- | --- | --- | --- | --- | --- | --- |
| **ORF#** | **Gene name /location /direction** | **No. of amino acids** | **G+C content(%)** | **Similar protein/strain/GenBank accession No.** | **% Identity/% Similarity (No. of aa overlap)** | |
| O10-01 | *rep* | 676 | 55.0 | DNA helicase Rep | 99/99(675) | |
|  | (1-2031) |  |  | [*Plesiomonas shigelloides*] | | |
|  | + |  |  | [WP_010862901](https://www.ncbi.nlm.nih.gov/protein/499149593?report=genbank&log$=protalign&blast_rank=1&RID=056S2JS8014) | | |
| O10-02 | *rfaH* | 136 | 52.0 | transcription/translation regulatory transformer protein RfaH | 95/97(133) | |
|  | (2186-2596) |  |  | [*Plesiomonas shigelloides*] | | |
|  | - |  |  | [WP_084977043](https://www.ncbi.nlm.nih.gov/protein/1183756428?report=genbank&log$=protalign&blast_rank=1&RID=056S2JS8014) | | |
| O10-03 | *c5* | 108 | 56.0 | Cytochrome c5 | 98/98(106) | |
|  | (2931-3257) |  |  | [*Plesiomonas shigelloides*] | | |
|  | - |  |  | [SBT59692](https://www.ncbi.nlm.nih.gov/protein/1037176416?report=genbank&log$=protalign&blast_rank=2&RID=076Z2GGR015) | | |
| O10-04 | *wzz* | 370 | 36.9 | Wzz | 83/89(325) | |
|  | (3898-5010) |  |  | [*Plesiomonas shigelloides*] | | |
|  | + |  |  | [AAG17407](https://www.ncbi.nlm.nih.gov/protein/10442654?report=genbank&log$=protalign&blast_rank=3&RID=076Z2GGR015) | | |
| O10-05 | *orf5* | 43 | 51.4 | hypothetical protein ATN83_p10004 (plasmid) | 72/79(31) | |
|  | (5474-5605) |  |  | [*Raoultella ornithinolytica*] | | |
|  | + |  |  | [ALQ49382](https://www.ncbi.nlm.nih.gov/protein/958289429?report=genbank&log$=protalign&blast_rank=1&RID=076Z2GGR015) | | |
| O10-06 | *wzx* | 453 | 39.1 | flippase | 79/88(399) | |
|  | (5859-7220) |  |  | [*Shewanella decolorationis*] | | |
|  | + |  |  | [WP_023267163](https://www.ncbi.nlm.nih.gov/protein/555469840?report=genbank&log$=protalign&blast_rank=1&RID=076Z2GGR015) | | |
| O10-07 | *ugd* | 388 | 50.4 | nucleotide sugar dehydrogenase | 94/97(380) | |
|  | (7271-8437) |  |  | [*Shewanella algae*] | | |
|  | + |  |  | [WP_071237813](https://www.ncbi.nlm.nih.gov/protein/1098557498?report=genbank&log$=protalign&blast_rank=1&RID=076Z2GGR015) | | |
| O10-08 | *wzy* | 381 | 36.7 | hypothetical protein | | 32/53(193) |
|  | (8517-9662) |  |  | [*Marinobacter sp. EN3*] | | |
|  | *+* |  |  | [WP_023010815](https://www.ncbi.nlm.nih.gov/protein/552557894?report=genbank&log$=protalign&blast_rank=1&RID=076Z2GGR015) | | |
| O10-09 | *GT* | 400 | 40.6 | glycosyltransferase family 2 protein | 40/61(215) | |
|  | (9664-10866) |  |  | [*Prosthecochloris sp. CIB 2401*] | | |
|  | *+* |  |  | [WP_068866863](https://www.ncbi.nlm.nih.gov/protein/1057574889?report=genbank&log$=protalign&blast_rank=1&RID=076Z2GGR015) | | |
| O10-10 | *GT* | 413 | 33.3 | glycosyl transferase group 1 protein | 35/52(220) | |
|  | (10863-12104) |  |  | [*Plesiomonas shigelloides*] | | |
|  | *+* |  |  | [WP_010862894](https://www.ncbi.nlm.nih.gov/protein/499149585?report=genbank&log$=protalign&blast_rank=28&RID=076Z2GGR015) | | |
| O10-11 | *orf11* | 74 | 72.7 | hypothetical protein | 43/63(19) | |
|  | (12086-12310) |  |  | [*Agromyces sp. CF514*] | | |
|  | *+* |  |  | [WP_092967418](https://www.ncbi.nlm.nih.gov/protein/1224951934?report=genbank&log$=protalign&blast_rank=2&RID=076Z2GGR015) | | |
| O10-12 | *GT* | 230 | 41.4 | glycosyl transferase | 88/92(213) | |
|  | (12590-13282) |  |  | [*Shewanella algae*] | | |
|  | *+* |  |  | [WP_071237911](https://www.ncbi.nlm.nih.gov/protein/1098557596?report=genbank&log$=protalign&blast_rank=1&RID=076Z2GGR015) | | |
| O10-13 | *galE* | 345 | 38.8 | UDP-glucose 4-epimerase | 94/97(337) | |
|  | (13303-14340) |  |  | [*Shewanella* sp. CG12_big_fil_rev_8_21_14_0_65_47_15] | | |
|  | *+* |  |  | [PIW59854](https://www.ncbi.nlm.nih.gov/protein/PIW59854.1?report=genbank&log$=protalign&blast_rank=13&RID=7H1XXBW8015) | | |
| O10-14 | *wecB* | 285 | 42.5 | UDP-N-acetylglucosamine 2-epimerase (non-hydrolyzing) | 96/97(278) | |
|  | (14469-15353) |  |  | [*Vibrio vulnificus*] | | |
|  | *+* |  |  | [WP_103194257](https://www.ncbi.nlm.nih.gov/protein/WP_103194257.1?report=genbank&log$=protalign&blast_rank=1&RID=74XMGN1S014) | | |
| O10-15 | *transposase* | 50 | 54.0 | transposase | 92/95(47) | |
|  | (15418-15570) |  |  | [*Pectobacterium carotovorum subsp. brasiliense*] | | |
|  | *+* |  |  | [KFF63539](https://www.ncbi.nlm.nih.gov/protein/671732151?report=genbank&log$=protalign&blast_rank=1&RID=076Z2GGR015) | | |
| O10-16 | *transposase* | 97 | 54.0 | Transposase | 92/92/(92) | |
|  | (15622-15915) |  |  | [*Plesiomonas shigelloides 302-73*] | | |
|  | + |  |  | [EON89274](https://www.ncbi.nlm.nih.gov/protein/499066958?report=genbank&log$=protalign&blast_rank=1&RID=076Z2GGR015) | | |
| O10-17 | *orf17* | 51 | 39.0 | No significant similarity found |  | |
|  | (16231-16386) |  |  |  | | |
|  | *+* |  |  |  | | |
| O10-18 | *orf18* | 39 | 46.0 | hypothetical protein VCS12_07095 | 54/72(27) | |
|  | (16983-17102) |  |  | [*Vibrio cholerae*] | | |
|  | *+* |  |  | [OEG78106](https://www.ncbi.nlm.nih.gov/protein/1068786972?report=genbank&log$=protalign&blast_rank=1&RID=076Z2GGR015) | | |
| O10-19 | *fnlA* | 345 | 38.8 | UDP-N-acetylglucosamine 4,6-dehydratase | 98/99(342) | |
|  | (17587-18624) |  |  | [*Plesiomonas shigelloides*] | | |
|  | *+* |  |  | [WP_010862887](https://www.ncbi.nlm.nih.gov/protein/499149577?report=genbank&log$=protalign&blast_rank=1&RID=076Z2GGR015) | | |
| O10-20 | *fnlB* | 368 | 38.1 | UDP-2-acetamido-2,6-dideoxy-beta-L-talose 4-dehydrogenase | 63/80(297) | |
|  | (18625-19731) |  |  | [*Escherichia coli*] | | |
|  | *+* |  |  | AAV74536 | | |
| O10-21 | *fnlC* | 370 | 42.5 | UDP-2-acetamio-2,6-dideoxy-L-talose 2-epimerase | 99/99(368) | |
|  | (19766-20878) |  |  | [*Plesiomonas shigelloides*] | | |
|  | *+* |  |  | [WP_010862885](https://www.ncbi.nlm.nih.gov/protein/499149575?report=genbank&log$=protalign&blast_rank=1&RID=076Z2GGR015) | | |
| O10-22 | *wbuB* | 420 | 40.9 | glycosyltransferase WbuB | 95/96(388) | |
|  | (20891-22153) |  |  | [*Plesiomonas shigelloides*] | | |
|  | *+* |  |  | WP_036769296 | | |
| O10-23 | *pglC* | 391 | 43.0 | DegT/DnrJ/EryC1/StrS aminotransferase family protein | 99/98(387) | |
|  | (23404-24579) |  |  | [*Plesiomonas shigelloides*] | | |
|  | *+* |  |  | [WP_010862880](https://www.ncbi.nlm.nih.gov/protein/499149570?report=genbank&log$=protalign&blast_rank=1&RID=076Z2GGR015) | | |
| O10-24 | *wbgZ* | 509 | 44.3 | wbgZ | 96/98(499) | |
|  | (25058-26587) |  |  | [*Shigella sonnei*] | | |
|  | + |  |  | [AGI19337](https://www.ncbi.nlm.nih.gov/protein/471846220?report=genbank&log$=protalign&blast_rank=7&RID=0V4NAS0G014) | | |
| O10-25 | *aqpZ* | 233 | 54.6 | aquaporin Z | 99/100(233) | |
|  | (26706-27407) |  |  | [*Plesiomonas shigelloides*] | | |
|  | + |  |  | [WP_039046419](https://www.ncbi.nlm.nih.gov/protein/743529336?report=genbank&log$=protalign&blast_rank=2&RID=076Z2GGR015) | | |

| **Table S3** Characteristics of the ORFs of *P. shigelloides* O12H35 O antigen gene cluster | | | | | |
| --- | --- | --- | --- | --- | --- |
| **ORF#** | **Gene name /location /direction** | **No. of amino acids** | **G+C content(%)** | **Similar protein/strain/GenBank accession No.** | **% Identity/% Similarity (No. of aa overlap)** |
| O12-01 | *rep* | 657 | 55.2 | ATP-dependent DNA helicase Rep | 99/99(656) |
|  | (1-1974) |  |  | [*Plesiomonas shigelloides*] | |
|  | + |  |  | WP_084977044 | |
| O12-02 | *rfaH* | 127 | 52.2 | transcription/translation regulatory transformer protein RfaH | 99/99(126) |
|  | (2129-2512) |  |  | [*Plesiomonas shigelloides*] | |
|  | - |  |  | WP_064977063 | |
| O12-03 | *c5* | 141 | 56.6 | cytochrome c5 family protein | 95/96(136) |
|  | (2875-3300) |  |  | *[Plesiomonas shigelloides*] | |
|  | - |  |  | WP_084977097 | |
| O12-04 | *orf4* | 399 | 37.0 | hypothetical protein | 44/65(228) |
|  | (4744-5943) |  |  | [*Pantoea agglomerans*] | |
|  | + |  |  | WP_089414010 | |
| O12-05 | *wbbN* | 295 | 42.6 | glycosyltransferase family 2 | 50/63(190) |
|  | (6997-7884) |  |  | [*Serratia liquefaciens*] | |
|  | + |  |  | WP_044549386 | |
| O12-06 | *glf* | 384 | 41.0 | UDP-galactopyranosemutase | 79/89(342) |
|  | (7881-9035) |  |  | [*Klebsiella pneumoniae*] | |
|  | + |  |  | WP_065799463 | |
| O12-07 | *wbbM* | 610 | 38.6 | WbbM | 67/81(499) |
|  | (9059-10891) |  |  | [*Klebsiella pneumonia* subsp. rhinoscleromatis SB3432] | |
|  | + |  |  | CCI75836 | |
| O12-08 | *wzt* | 214 | 38.0 | ABC transporter ATP-binding protein | 90/96(205) |
|  | (11056-11700) |  |  | [*Salmonella enterica*] | |
|  | + |  |  | WP_094882928 | |
| O12-09 | *wzm* | 300 | 34.9 | ABC transporter permease | 89/94(240) |
|  | (11802-12704) |  |  | [*Salmonella enterica*] | |
|  | + |  |  | WP_094882927 | |
| O12-10 | *wbbO* | 395 | 35.4 | glycosyltransferase family 1 protein | 57/75(283） |
|  | (12749-13936) |  |  | [*Serratia* sp. Leaf50] | |
|  | + |  |  | WP_055780316 | |
| O12-11 | *aqpZ* | 233 | 54.6 | aquaporin Z | 99/99(232) |
|  | (15825-16526) |  |  | [*Plesiomonas shigelloides*] | |
|  | + |  |  | WP_039046419 | |

| **Table S4** Characteristics of the ORFs of *P. shigelloides* O23H1a1C O antigen gene cluster | | | | | |
| --- | --- | --- | --- | --- | --- |
| **ORF#** | **Gene name /location /direction** | **No. of amino acids** | **G+C content(%)** | **Similar protein/strain/GenBank accession No.** | **% Identity/% Similarity (No. of aa overlap)** |
| O23-01 | *rep* | 676 | 55.0 | DNA helicase Rep | 100/100(676) |
|  | (1-2031) |  |  | [*Plesiomonas shigelloides*] | |
|  | + |  |  | WP_010862901 | |
| O23-02 | *rfaH* | 136 | 52.0 | transcription/translation regulatory transformer protein RfaH | 98/99(135) |
|  | (2187-2597) |  |  | [*Plesiomonas shigelloides*] | |
|  | - |  |  | WP_084977043 | |
| O23-03 | *c5* | 141 | 56.2 | cytochrome c5 | 99/99(140) |
|  | (2934-3359) |  |  | [*Plesiomonas shigelloides* 302-73] | |
|  | - |  |  | EON89259 | |
| O23-04 | *orf4* | 44 | 42.5 | No significant similarity found | |
|  | (3732-3878) |  |  |  | |
|  | + |  |  |  | |
| O23-05 | *wzz* | 372 | 36.9 | Wzz | 79/88(326) |
|  | (3906-5024) |  |  | [*Plesiomonas shigelloides*] | |
|  | + |  |  | AAG17407 | |
| O23-06 | *wecA* | 338 | 40.7 | undecaprenyl-phosphate alpha-N-acetylglucosaminyl 1-phosphate transferase | 93/96(321) |
|  | (5099-6115) |  |  | [*Plesiomonas shigelloides*] | |
|  | + |  |  | WP_084977041 | |
| O23-07 | *mnaA* | 358 | 43.3 | UDP-N-acetylglucosamine 2-epimerase (non-hydrolyzing) | 81/89(317) |
|  | (6461-7537) |  |  | [*Escherichia coli*] | |
|  | + |  |  | WP_040079036 | |
| O23-08 | *GT* | 352 | 29.7 | glycosyltransferase family 4 protein | 42/59(211) |
|  | (7543-8601) |  |  | [*Vibrio cholerae*] | |
|  | + |  |  | WP_001082407 | |
| O23-09 | *wzy* | 392 | 29.0 | Wzy | 29/49(186) |
|  | (8577-9755) |  |  | [*Salmonella enterica*] | |
|  | + |  |  | ABL63455 | |
| O23-10 | *wzx* | 406 | 34.4 | putative O antigen flippase | 27/48(185) |
|  | (9742-10962) |  |  | [*Escherichia coli* S88] | |
|  | + |  |  | CAN87668 | |
| O23-11 | *orf11* | 39 | 40.9 | hypothetical protein AUJ18_01345 | 48/70(22) |
|  | (11451-11570) |  |  | [*Candidatus Hydrogenedentes bacterium* CG1_02_42_14] | |
|  | + |  |  | OIO34692 | |
| O23-12 | *GT* | 133 | 28.1 | glycosyltransferase | 49/69(87) |
|  | (11657-12058) |  |  | [*Vibrio cholerae*] | |
|  | + |  |  | WP_000656434 | |
| O23-13 | *purD* | 435 | 36.5 | phosphoribosylamine--glycine ligase | 33/50(222) |
|  | (12055-13362) |  |  | [*Vibrio parahaemolyticus*] | |
|  | + |  |  | WP_053805965 | |
| O23-14 | *transposase* | 99 | 55.4 | putative transposase | 91/92(91) |
|  | (13898-14197) |  |  | [*Klebsiella pneumoniae*] | |
|  | + |  |  | SMA25156 | |
| O23-15 | *insH* | 99 | 55.7 | transposase insH for insertion sequence element IS5Y, partial | 91/92(92) |
|  | (14214-14513) |  |  | [*Escherichia coli* KTE64] | |
|  | + |  |  | EOV68461 | |
| O23-16 | *orf16* | 44 | 52.5 | hypothetical protein PPECC9_2050, partial | 76/85(36) |
|  | (14517-14651) |  |  | [*Escherichia coli* PCN009] | |
|  | + |  |  | OAF98265 | |
| O23-17 | *aqpZ* | 233 | 54.6 | aquaporin Z | 99/100(233) |
|  | (14805-15506) |  |  | [*Plesiomonas shigelloides*] | |
|  | + |  |  | WP_039046419 | |

| **Table S5** Characteristics of the ORFs of *P. shigelloides* O25H3 O antigen gene cluster | | | | | | |
| --- | --- | --- | --- | --- | --- | --- |
| **ORF#** | **Gene name /location /direction** | **No. of amino acids** | **G+C content(%)** | **Similar protein/strain/GenBank accession No.** | | **% Identity/% Similarity (No. of aa overlap)** |
| O25-01 | *rep* | 676 | 55.2 | ATP-dependent DNA helicase Rep | | 99/100(676) |
|  | (1-2031) |  |  | [*Plesiomonas shigelloides*] | | |
|  | + |  |  | WP_084977044 | | |
| O25-02 | *rfaH* | 158 | 52.0 | transcription/translation regulatory transformer protein RfaH | | 96/97(154) |
|  | (2186-2662) |  |  | [*Plesiomonas shigelloides*] | | |
|  | - |  |  | WP_084977043 | | |
| O25-03 | *c5* | 108 | 56.2 | cytochrome c5 | | 97/98(106) |
|  | (2937-3263) |  |  | [*Plesiomonas shigelloides* 302-73] | | |
|  | - |  |  | EON89259 | | |
| O25-04 | *GT* | 749 | 37.0 | MULTISPECIES: glycosyl transferase family 1 | 54/72(538) | |
|  | (3937-6186) |  |  | [*Enterobacteriaceae*] | | |
|  | + |  |  | WP_001048111 | | |
| O25-05 | *wzm* | 259 | 33.8 | MULTISPECIES: O89/O101/O162 family O-antigen ABC transporter permease subunit Wzm | | 83/95(247) |
|  | (6183-6962) |  |  | [*Enterobacteriaceae*] | | |
|  | + |  |  | WP_000665069 | | |
| O25-06 | *wzt* | 250 | 39.0 | MULTISPECIES: O89/0101/0162 family O-antigen ABC transporter ATP-binding protein Wzt | | 89/95(239) |
|  | (6964-7716) |  |  | [*Enterobacteriaceae*] | | |
|  | + |  |  | WP_000018741 | | |
| O25-07 | *GT* | 347 | 40.5 | glycosyl transferase, group 1 | | 56/72(247) |
|  | (7776-8819) |  |  | [*Citrobacter europaeus*] | | |
|  | + |  |  | SBW26874 | | |
| O25-08 | *rfpB* | 381 | 35.5 | glycosyltransferase family 1 protein | | 67/82(309) |
|  | (8836-9981) |  |  | [*Citrobacter europaeus*] | | |
|  | + |  |  | WP_087051356 | | |
| O25-09 | *methyltransferase* | 420 | 34.9 | class I SAM-dependent methyltransferase | | 49/69(297) |
|  | (10091-11353) |  |  | [*Citrobacter europaeus*] | | |
|  | + |  |  | [WP_087052556](https://www.ncbi.nlm.nih.gov/protein/WP_087052556.1?report=genbank&log$=protalign&blast_rank=1&RID=75061827014) | | |
| O25-10 | *GT* | 679 | 34.4 | glycosyl transferase family 1 | | 67/79(550) |
|  | (11358-13397) |  |  | [*Citrobacter europaeus*] | | |
|  | + |  |  | WP_087052557 | | |
| O25-11 | *foxred* | 371 | 47.6 | FAD-binding oxidoreductase | | 73/84(315) |
|  | (13463-14578) |  |  | [*Uliginosibacterium gangwonense*] | | |
|  | + |  |  | WP_026258713 | | |
| O25-12 | *GT* | 319 | 40.9 | glycosyl transferase family 2 | | 65/80(256) |
|  | (14645-15604) |  |  | [*Serratia* sp. DD3] | | |
|  | + |  |  | WP_037386144 | | |
| O25-13 | *oxidoreductase* | 258 | 56.1 | NAD(P)-dependent oxidoreductase | | 73/83(214) |
|  | (15661-16437) |  |  | [*Duganella sp*. Root336D2] | | |
|  | + |  |  | [WP_055938288](https://www.ncbi.nlm.nih.gov/protein/WP_055938288.1?report=genbank&log$=protalign&blast_rank=1&RID=750F5AKY014) | | |
| O25-14 | *xylA* | 276 | 54.1 | xylose isomerase | | 64/78(212) |
|  | (16455-17285) |  |  | [*Escherichia coli*] | | |
|  | + |  |  | KFH90141 | | |
| O25-15 | *GT* | 718 | 54.8 | glycosyltransferase family 2 protein | | 77/86(230) |
|  | (17640-19796) |  |  | [*Duganella* sp. Root336D2] | | |
|  | + |  |  | WP_055938280 | | |
| O23-16 | *aqpZ* | 233 | 54.6 | aquaporin Z | | 99/100(233) |
|  | (19981-20682) |  |  | [*Plesiomonas shigelloides*] | | |
|  | + |  |  | WP_039046419 | | |

| **Table S6** Characteristics of the ORFs of *P. shigelloides* O26H1a1c O antigen gene cluster | | | | | |
| --- | --- | --- | --- | --- | --- |
| **ORF#** | **Gene name /location /direction** | **No. of amino acids** | **G+C content(%)** | **Similar protein/strain/GenBank accession No.** | **% Identity/% Similarity (No. of aa overlap)** |
| O26-01 | *rep* | 676 | 55.2 | DNA helicase Rep | 99/99(675) |
|  | (1-2031) |  |  | [*Plesiomonas shigelloides*] | |
|  | + |  |  | WP_039046404 | |
| O26-02 | *rfaH* | 158 | 51.8 | transcription/translation regulatory transformer protein RfaH | 98/99(157) |
|  | (2186-2662) |  |  | [*Plesiomonas shigelloides*] | |
|  | + |  |  | WP_039046405 | |
| O26-03 | *c5* | 108 | 56.2 | cytochrome c5 | 99/100(108) |
|  | (2936-3262) |  |  | [*Plesiomonas shigelloides* 302-73] | |
|  | + |  |  | EON89259 | |
| O26-04 | orf4 | 51 | 46.0 | No significant similarity found |  |
|  | (3462-3617) |  |  |  | |
|  | + |  |  |  | |
| O26-05 | *wzz* | 375 | 36.9 | Wzz | 90/92(339) |
|  | (3906-5033) |  |  | [*Plesiomonas shigelloides*] | |
|  | + |  |  | AAG17407 | |
| O26-06 | *rmlB* | 338 | 48.0 | dTDP-glucose 4,6-dehydratase | 89/95(323) |
|  | (5234-6250) |  |  | [*Vibrio cholerae*] | |
|  | + |  |  | WP_029628153 | |
| O26-07 | *rmlA* | 260 | 46.8 | glucose-1-phosphate thymidylyltransferase | 95/96(252) |
|  | (6371-7153) |  |  | [*Vibrio cholerae*] | |
|  | + |  |  | WP_000676091 | |
| O26-08 | *rmlD* | 273 | 48.5 | RmlD | 85/93(254) |
|  | (7219-8040) |  |  | [*Vibrio anguillarum*] | |
|  | + |  |  | AAZ66344 | |
| O26-09 | *rmlC* | 178 | 40.2 | RmlC | 83/88(158) |
|  | (8040-8576) |  |  | [*Vibrio anguillarum*] | |
|  | + |  |  | AAZ66345 | |
| O26-10 | *wchZ* | 228 | 33.3 | nucleotidyltransferaseWchZ | 37/66(103) |
|  | (9090-9776) |  |  | [*Streptococcus pneumoniae*] | |
|  | + |  |  | CGF63361 | |
| O26-11 | *HAD family hydrolase* | 212 | 32.2 | HAD family hydrolase, partial | 58/78(165) |
|  | (9896-10534) |  |  | [*Escherichia coli*] | |
|  | + |  |  | WP_097742066 | |
| O26-12 | *wzx* | 408 | 34.5 | Wzx | 36/60(211) |
|  | (11172-12398） |  |  | [*Escherichia coli*] | |
|  | + |  |  | ABF01008 | |
| O26-13 | *tagB* | 398 | 27.4 | glycosyl/glycerophosphatetransferase involved in teichoic acid biosynthesis TagF/TagB/EpsJ/RodC | 35/55(223) |
|  | (12884-14080) |  |  | [*Bacillus thuringiensis*] | |
|  | + |  |  | WP_023523538 | |
| O26-14 | *orf14* | 37 | 38.6 | No significant similarity found |  |
|  | (14592-14705) |  |  |  | |
|  | + |  |  |  | |
| O26-15 | *orf15* | 48 | 43.0 | No significant similarity found |  |
|  | (15017-15163) |  |  |  | |
|  | + |  |  |  | |
| O26-16 | *wzy* | 298 | 62.0 | oligosaccharide repeat unit polymerase | 36/56(169) |
|  | (15231-16127) |  |  | [*Flavobacterium sp*. GSP27] | |
|  | + |  |  | [WP_126555327](https://www.ncbi.nlm.nih.gov/protein/WP_126555327.1?report=genbank&log$=protalign&blast_rank=1&RID=750UJPFG015) | |
| O26-17 | *rfbF* | 291 | 32.5 | dTDP-rhamnosyltransferaseRfbF | 38/55(143) |
|  | (16120-16995) |  |  | [*Shigella flexneri*] | |
|  | + |  |  | WP_001087598 | |
| O26-18 | *orf18* | 41 | 51.0 | No significant similarity found |  |
|  | (17457-17582) |  |  |  | |
|  | + |  |  |  | |
| O26-19 | *wbuX* | 378 | 43.5 | WbuX (WbeC) | 77/89(328) |
|  | (17728-18864) |  |  | [*Yersinia ruckeri*ATCC 29473] | |
|  | + |  |  | EEP97747 | |
| O26-20 | *orf20* | 45 | 47.1 | No significant similarity found |  |
|  | (19204-19341) |  |  |  | |
|  | + |  |  |  | |
| O26-21 | *orf21* | 38 | 39.8 | No significant similarity found |  |
|  | (19342-19458) |  |  |  | |
|  | + |  |  |  | |
| O26-22 | *hisF* | 261 | 60.0 | imidazole glycerol phosphate synthase subunit HisF | 70/82(215) |
|  | (19482-20267) |  |  | [*Burkholderiales bacterium* PBB2] | |
|  | + |  |  | OYU27708 | |
| O26-23 | *fnlA* | 326 | 40.4 | UDP-N-acetylglucosamine 4,6-dehydratase | 91/95(312) |
|  | (20342-21322) |  |  | [*Vibrio cholerae*] | |
|  | + |  |  | ADF80963 | |
| O26-24 | *fnlB* | 368 | 38.1 | FnlB | 63/80(294) |
|  | (21323-22429) |  |  | [*Escherichia coli*] | |
|  | + |  |  | AAV74536 | |
| O26-25 | *fnlC* | 376 | 44.4 | UDP-N-acetylglucosamine 2-epimerase (non-hydrolyzing) | 97/98(369) |
|  | (22446-23576) |  |  | [*Vibrio mimicus*] | |
|  | + |  |  | WP_000734417 | |
| O26-26 | *wbuB* | 401 | 36.1 | WbuB | 89/94(377) |
|  | (23580-24785) |  |  | [*Vibrio cholerae*] | |
|  | + |  |  | ADF80966 | |
| O26-27 | *wbhP* | 320 | 38.7 | UDP-glucose 4-epimerase | 75/87(276) |
|  | (24788-25750) |  |  | [*Vibrio mimicus*] | |
|  | + |  |  | WP_000866329 | |
| O26-28 | *wcaJ* | 184 | 41.3 | sugar transferase | 90/94(173) |
|  | (25747-26301) |  |  | [*Vibrio cholerae*] | |
|  | + |  |  | WP_054103950 | |
| O26-29 | *wbgZ* | 639 | 44.3 | wbgZ | 88/92(588) |
|  | (26385-28304) |  |  | [*Shigella sonnei*] | |
|  | + |  |  | AGI19337 | |
| O26-30 | *aqpZ* | 233 | 54.6 | aquaporin Z | 99/99(232) |
|  | (28423-29124) |  |  | [*Plesiomonas shigelloides*] | |
|  | + |  |  | WP_039046419 | |

| **Table S7** Characteristics of the ORFs of *P. shigelloides* O32H37 O antigen gene cluster | | | | | |
| --- | --- | --- | --- | --- | --- |
| **ORF#** | **Gene name /location /direction** | **No. of amino acids** | **G+C content(%)** | **Similar protein/strain/GenBank accession No.** | **% Identity/% Similarity (No. of aa overlap)** |
| O32-01 | *rep* | 676 | 55.0 | DNA helicase Rep | 100/100(676) |
|  | (1-2031) |  |  | [*Plesiomonas shigelloides*] | |
|  | + |  |  | WP_010862901 | |
| O32-02 | *rfaH* | 136 | 52.2 | transcription/translation regulatory transformer protein RfaH | 97/100(136) |
|  | (2187-2597) |  |  | [*Plesiomonas shigelloides*] | |
|  | + |  |  | WP_064977063 | |
| O32-03 | *c5* | 108 | 56.2 | cytochrome c5 | 98/99(107) |
|  | (2949-3275) |  |  | [*Plesiomonas shigelloides* 302-73] | |
|  | + |  |  | EON89259 | |
| O32-04 | *wzz* | 358 | 36.9 | Wzz | 40/56(208) |
|  | (3939-5015) |  |  | [*Plesiomonas shigelloides*] | |
|  | + |  |  | AAG17407 | |
| O32-05 | *gne* | 317 | 33.0 | Gne | 64/78(248) |
|  | (5089-6042) |  |  | [*Yersinia pseudotuberculosis*] | |
|  | + |  |  | AKA21005 | |
| O32-06 | *exopolysaccharide* | 299 | 48.6 | exopolysaccharide biosynthesis protein | 74/84(253) |
|  | (6074-6973) |  |  | [*Aeromonas caviae*] | |
|  | + |  |  | KOG94764 | |
| O32-07 | *GT* | 338 | 31.9 | glycosyltransferase | 57/73(248) |
|  | (6970-7986) |  |  | [*Salmonella enterica*] | |
|  | + |  |  | AFW04728 | |
| O32-08 | *wzx* | 412 | 27.9 | O166 family O-antigen flippase | 45/65(259) |
|  | (8127-9365) |  |  | [*Escherichia coli*] | |
|  | + |  |  | WP_033815058 | |
| O32-09 | *GT* | 274 | 28.4 | glycosyltransferase family 2 protein | 57/73(199) |
|  | (9375-10199) |  |  | [*Proteus mirabilis*] | |
|  | + |  |  | WP_063691847 | |
| O32-10 | *GT* | 374 | 34.3 | glycosyltransferase family 4 protein | 58/79(285) |
|  | (10160-11284) |  |  | [*Edwardsiella tarda*] | |
|  | + |  |  | WP_074428711 | |
| O32-11 | *wzy* | 382 | 26.7 | O-antigen polymerase | 33/53(200) |
|  | (11315-12463) |  |  | [*Salmonella enterica*] | |
|  | + |  |  | AFW04828 | |
| O32-12 | *GT* | 362 | 48.1 | glycosyl transferase | 63/78(284) |
|  | (12466-13554) |  |  | [*Shewanella* sp. SACH] | |
|  | + |  |  | WP_071939653 | |
| O32-13 | *aqpZ* | 233 | 54.6 | aquaporin Z | 99/100(233) |
|  | (13729-14430) |  |  | [*Plesiomonas shigelloides*] | |
|  | + |  |  | WP_039046419 | |

| **Table S8** Characteristics of the ORFs of *P. shigelloides* O33H38 O antigen gene cluster | | | | | | |
| --- | --- | --- | --- | --- | --- | --- |
| **ORF#** | **Gene name /location /direction** | **No. of amino acids** | **G+C content(%)** | **Similar protein/strain/GenBank accession No.** | **% Identity/% Similarity (No. of aa overlap)** | |
| O33-01 | *rep* | 676 | 55.2 | DNA helicase Rep | 99/99(674) | |
|  | (1-2031) |  |  | [*Plesiomonas shigelloides*] | | |
|  | + |  |  | [WP_039046404](https://www.ncbi.nlm.nih.gov/protein/743529321?report=genbank&log$=protalign&blast_rank=3&RID=172AN539015) | | |
| O33-02 | *rfaH* | 158 | 51.8 | transcription/translation regulatory transformer protein RfaH | 97/99(157) | |
|  | (2185-2661) |  |  | [*Plesiomonas shigelloides*] | | |
|  | - |  |  | [WP_039046405](https://www.ncbi.nlm.nih.gov/protein/743529322?report=genbank&log$=protalign&blast_rank=1&RID=172AN539015) | | |
| O33-03 | *c5* | 108 | 56.2 | cytochrome c5 | 99/99(107) | |
|  | (2935-3261) |  |  | [*Plesiomonas shigelloides 302-73*] | | |
|  | - |  |  | [EON89259](https://www.ncbi.nlm.nih.gov/protein/499066942?report=genbank&log$=protalign&blast_rank=1&RID=172AN539015) | | |
| O33-04 | *wzz* | 368 | 36.9 | Wzz | 91/94(343) | |
|  | (3900-5006) |  |  | [*Plesiomonas shigelloides*] | | |
|  | + |  |  | [AAG17407](https://www.ncbi.nlm.nih.gov/protein/10442654?report=genbank&log$=protalign&blast_rank=1&RID=18HMXHG8013) | | |
| O33-05 | *wbgT/gna* | 426 | 36.0 | WbgT | 83/92(394) | |
|  | (5547-6827) |  |  | [*Plesiomonas shigelloides*] | | |
|  | + |  |  | [AAG17408](https://www.ncbi.nlm.nih.gov/protein/10442655?report=genbank&log$=protalign&blast_rank=21&RID=18HMXHG8013) | | |
| O33-06 | *vipB* | 338 | 47.2 | Vi polysaccharide biosynthesis UDP-N-acetylglucosaminuronic acid C-4 epimerase TviC | 79/88(297) | |
|  | (6935-7951) |  |  | [*Vibrio vulnificus*] | | |
|  | + |  |  | [WP_045623841](https://www.ncbi.nlm.nih.gov/protein/782734092?report=genbank&log$=protalign&blast_rank=1&RID=1JEUWB80014) | | |
| O33-07 | *wzx* | 432 | 29.8 | putative O-antigen flippase | 38/62(272) | |
|  | (7952-9250) |  |  | [*Escherichia coli*] | | |
|  | + |  |  | [ABB04485](https://www.ncbi.nlm.nih.gov/protein/77862418?report=genbank&log$=protalign&blast_rank=3&RID=1HN4T79B014) | | |
| O33-08 | *wzy* | 414 | 28.6 | oligosaccharide repeat unit polymerase | | 46/70(287) |
|  | (9269-10513) |  |  | [*Moritella viscosa*] | | |
|  | *+* |  |  | [WP_075532677](https://www.ncbi.nlm.nih.gov/protein/1129585116?report=genbank&log$=protalign&blast_rank=1&RID=18HMXHG8013) | | |
| O33-09 | *wbuB* | 376 | 57.7 | glycosyltransferase WbuB | 23/47(151) | |
|  | (10531-11661) |  |  | [*Pseudomonas fluorescens*] | | |
|  | *+* |  |  | [WP_042728442](https://www.ncbi.nlm.nih.gov/protein/757175850?report=genbank&log$=protalign&blast_rank=12&RID=1G04MZ3D014) | | |
| O33-10 | *GT* | 371 | 33.0 | glycosyltransferase family 1 protein | 76/87(322) | |
|  | (11746-12861) |  |  | [*Aeromonas fluvialis*] | | |
|  | *+* |  |  | [WP_084202210](https://www.ncbi.nlm.nih.gov/protein/1180717074?report=genbank&log$=protalign&blast_rank=1&RID=18PDK4ZT013) | | |
| O33-11 | *fnlA* | 345 | 44.7 | UDP-glucose 4-epimerase | 93/96(333) | |
|  | (12854-13891) |  |  | [*Escherichia coli*] | | |
|  | *+* |  |  | [WP_052935822](https://www.ncbi.nlm.nih.gov/protein/920019503?report=genbank&log$=protalign&blast_rank=1&RID=18PDK4ZT013) | | |
| O33-12 | *qnlA* | 283 | 33.3 | QnlA | 69/81(229) | |
|  | (13924-14775) |  |  | [*Escherichia coli*] | | |
|  | *+* |  |  | [ABB04490](https://www.ncbi.nlm.nih.gov/protein/77862423?report=genbank&log$=protalign&blast_rank=10&RID=18PDK4ZT013) | | |
| O33-13 | *qnlB* | 371 | 37.9 | UDP-N-acetylglucosamine 2-epimerase (non-hydrolyzing) | 88/94(349) | |
|  | (14781-15896) |  |  | [*Escherichia coli* ] | | |
|  | *+* |  |  | [WP_053884006](https://www.ncbi.nlm.nih.gov/protein/928823703?report=genbank&log$=protalign&blast_rank=12&RID=1HN4T79B014) | | |
| O33-14 | *wbuB* | 396 | 32.3 | glycosyltransferase WbuB | 74/86(334) | |
|  | (15862-17052) |  |  | [*Shewanella xiamenensis*] | | |
|  | *+* |  |  | [WP_099458416](https://www.ncbi.nlm.nih.gov/protein/1273063113?report=genbank&log$=protalign&blast_rank=1&RID=18PDK4ZT013) | | |
| O33-15 | *galE* | 305 | 40.6 | UDP-glucose 4-epimerase | 61/77(232) | |
|  | (17063-17980) |  |  | [*Vibrio cholerae*] | | |
|  | *+* |  |  | [WP_046127202](https://www.ncbi.nlm.nih.gov/protein/806463929?report=genbank&log$=protalign&blast_rank=24&RID=1HN4T79B014) | | |
| O33-16 | *wbgZ* | 509 | 44.3 | wbgZ | 96/98/(500) | |
|  | (19014-20543) |  |  | [*Shigella sonnei*] | | |
|  | + |  |  | [AGI19337](https://www.ncbi.nlm.nih.gov/protein/471846220?report=genbank&log$=protalign&blast_rank=7&RID=18PDK4ZT013) | | |
| O33-17 | *aqpZ* | 233 | 54.6 | aquaporin Z | 99/99/(232) | |
|  | (20661-21362) |  |  | [*Plesiomonas shigelloides*] | | |
|  | *+* |  |  | [WP_039046419](https://www.ncbi.nlm.nih.gov/protein/743529336?report=genbank&log$=protalign&blast_rank=2&RID=18PDK4ZT013) | | |

| **Table S9** Characteristics of the ORFs of *P. shigelloides* O34H34 O antigen gene cluster | | | | | |
| --- | --- | --- | --- | --- | --- |
| **ORF#** | **Gene name /location /direction** | **No. of amino acids** | **G+C content(%)** | **Similar protein/strain/GenBank accession No.** | **% Identity/% Similarity (No. of aa overlap)** |
| O34-01 | *rep* | 676 | 55.0 | DNA helicase Rep | 100/100(676) |
|  | (1-2031) |  |  | [*Plesiomonas shigelloides*] | |
|  | + |  |  | WP_010862901 | |
| O34-02 | *rfaH* | 138 | 51.8 | transcription/translation regulatory transformer protein RfaH | 98/100(138) |
|  | (2185-2601) |  |  | [*Plesiomonas shigelloides*] | |
|  | + |  |  | WP_039046405 | |
| O34-03 | *c5* | 141 | 56.2 | cytochrome c5 | 97/98(139) |
|  | (2947-3372) |  |  | [*Plesiomonas shigelloides* 302-73] | |
|  | + |  |  | EON89259 | |
| O34-04 | *wzz* | 392 | 36.9 | Wzz | 41/57(210) |
|  | (3848-5026) |  |  | [*Plesiomonas shigelloides*] | |
|  | + |  |  | AAG17407 | |
| O34-05 | *oxidoreductase* | 346 | 43.7 | gfo/Idh/MocA family oxidoreductase | 83/93(346) |
|  | (5049-6089) |  |  | *[Vibrio harveyi]* | |
|  | + |  |  | EEO03407 | |
| O34-06 | *vipA* | 424 | 40.2 | Vi polysaccharide biosynthesis protein VipA/TviB | 84/90(383) |
|  | (6111-7385) |  |  | [*Aeromonas veronii*] | |
|  | + |  |  | OCQ45093 | |
| O34-07 | *wbbJ* | 145 | 45.3 | N-acetyltransferase | 92/95(137) |
|  | (7605-8042) |  |  | [*Aeromonas hydrophila*] | |
|  | + |  |  | WP_065476729 | |
| O34-08 | *wecE* | 358 | 42.5 | DegT/DnrJ/EryC1/StrS family aminotransferase | 87/94(333) |
|  | (8066-9142) |  |  | [*Vibrio cholerae*] | |
|  | + |  |  | WP_042988074 | |
| O34-09 | *orf9* | 396 | 49.6 | hypothetical protein BM485_17020 | 35/51(194) |
|  | (9147-10337) |  |  | [*Desulfobulbaceae bacterium DB1*] | |
|  | + |  |  | OKY73758 | |
| O34-10 | *wbpC* | 594 | 53.1 | WbpC | 41/57(345) |
|  | (10397-12181) |  |  | [*Pseudomonas aeruginosa* PAO1] | |
|  | + |  |  | AAC45854 | |
| O34-11 | *wzx* | 389 | 69.9 | O-antigen flippase Wzx | 36/57(116) |
|  | (12367-13536) |  |  | [*Janthinobacterium* sp. CG23_2] | |
|  | + |  |  | CUI08904 | |
| O34-12 | *wzy* | 428 | 42.9 | B-band O-antigen polymerase | 35/55(234) |
|  | (13539-14825) |  |  | [*Pseudomonas* sp. BAY1663] | |
|  | + |  |  | EXF44011 | |
| O34-13 | *hisF* | 254 | 38.2 | imidazole glycerol phosphate synthase subunit HisF | 87/94(239) |
|  | (15444-16208) |  |  | [*Vibrio cholerae*] | |
|  | + |  |  | WP_069647659 | |
| O34-14 | *wbpG* | 381 | 29.0 | LPS biosynthesis protein WbpG | 65/80(307) |
|  | (16215-17360) |  |  | [*Arcobacter butzleri* JV22] | |
|  | + |  |  | EFU70167 | |
| O34-15 | *wbpH* | 371 | 46.6 | glycosyltransferase WbpH | 57/75(276) |
|  | (17341-18456) |  |  | [*Pseudomonas* sp. CFII68] | |
|  | + |  |  | WP_018602988 | |
| O34-16 | *mnaA* | 353 | 47.6 | UDP-N-acetylglucosamine 2-epimerase (non-hydrolyzing) | 89/92(325) |
|  | (18468-19529) |  |  | [*Vibrio cholerae*] | |
|  | + |  |  | WP_069647662 | |
| O34-17 | *wbuB* | 404 | 37.4 | glycosyltransferase WbuB | 55/74(305) |
|  | (19558-20772) |  |  | [*Photorhabdus luminescens*] | |
|  | + |  |  | WP_065388901 | |
| O34-18 | *wbgX* | 383 | 37.4 | WbgX | 97/98(376) |
|  | (20774-21925) |  |  | [*Plesiomonas shigelloides*] | |
|  | + |  |  | AAG17414 | |
| O34-19 | *wbgZ* | 496 | 44.3 | wbgZ | 98/99(492) |
|  | (22976-24466) |  |  | [*Shigella sonnei*] | |
|  | + |  |  | AGI19337 | |
| O34-20 | *aqpZ* | 233 | 54.6 | aquaporin Z | 99/100(233) |
|  | (24585-25286) |  |  | [*Plesiomonas shigelloides*] | |
|  | + |  |  | WP_039046419 | |

| **Table S10** Characteristics of the ORFs of *P. shigelloides* O66H3 O antigen gene cluster | | | | | | |
| --- | --- | --- | --- | --- | --- | --- |
| **ORF#** | **Gene name /location /direction** | **No. of amino acids** | **G+C content(%)** | **Similar protein/strain/GenBank accession No.** | **% Identity/% Similarity (No. of aa overlap)** | |
| O66-01 | *rep* | 676 | 55.2 | ATP-dependent DNA helicase Rep | 99/100(676) | |
|  | (1-2031) |  |  | [*Plesiomonas shigelloides*] | | |
|  | + |  |  | WP_084977044 | | |
| O66-02 | *rfaH* | 136 | 52.0 | transcription/translation regulatory transformer protein RfaH | 96/98(134) | |
|  | (2185-2595) |  |  | [*Plesiomonas shigelloides*] | | |
|  | - |  |  | WP_084977043 | | |
| O66-03 | *c5* | 141 | 56.6 | cytochrome c5 family protein | 99/100(141) | |
|  | (2948-3373) |  |  | [*Plesiomonas shigelloides*] | | |
|  | - |  |  | WP_084977097 | | |
| O66-04 | *rmlB* | 361 | 55.9 | dTDP-glucose 4,6-dehydratase | 91/95 (343) | |
|  | (4057-5142) |  |  | [*Aeromonas caviae*] | | |
|  | + |  |  | OCW45355 | | |
| O66-05 | *rmlD* | 295 | 59.8 | dTDP-4-dehydrorhamnose reductase | 94/96 (284) | |
|  | (5142-6029) |  |  | [*Aeromonas hydrophil*a] | | |
|  | + |  |  | WP_045789382 | | |
| O66-06 | *rmlA* | 292 | 54.6 | glucose-1-phosphate thymidylyltransferaseRfbA | 98/97 (286) | |
|  | (6142-7020) |  |  | [*Aeromonassp. HZM*] | | |
|  | + |  |  | WP_043155072 | | |
| O66-07 | *wzm* | 271 | 32.0 | sugar ABC transporter permease | 52/71 (188) | |
|  | (7633-8448) |  |  | [*Citrobacter freundii*] | | |
|  | + |  |  | APR34022 | | |
| O66-08 | *wzt*  (8438-9751) | 437 | 37.9 | ABC transporter ATP-binding protein  [*Legionella santicrucis*] | | 58/75(332) |
|  | + |  |  | WP_058513537 | | |
| O66-09 | *methyltransferase* | 1115 | 45.5 | methyltransferase, partial | 81/89 (980) | |
|  | (9744-13091) |  |  | [*Aeromonas veronii*] | | |
|  | + |  |  | WP_011111115 | | |
| O66-10 | *wbsX* | 441 | 43.7 | Glycosyltransferase WbsX | 63/81(359) | |
|  | (13106-14431) |  |  | [*Nitrosomonas europaea*] | | |
|  | + |  |  | SDW71218 | | |
| O66-11 | *GT* | 361 | 38.2 | glycosyltransferase | 81/88 (319) | |
|  | (14796-15881) |  |  | [*Aeromonas hydrophila*] | | |
|  | + |  |  | AID71050 | | |
| O66-12 | *GT* | 264 | 41.6 | glycosyltransferase family 2 protein | 75/85(225) | |
|  | (15887-16681) |  |  | [*Shigella boydii*] | | |
|  | + |  |  | WP_096106369 | | |
| O66-13 | *wbhP* | 313 | 44.5 | NAD-dependent dehydratase | 50/66(203) | |
|  | (16681-17622) |  |  | [*Vibrio cholerae*] | | |
|  | + |  |  | WP_069212367 | | |
| O66-14 | *orf14* | 39 | 41.8 | No significant similarity found | | |
|  | (17678-17797) |  |  |  | | |
|  | + |  |  |  | | |
| O66-15 | *wbgZ* | 403 | 44.3 | wbgZ | 98/99(399) | |
|  | (18999-20210) |  |  | [*Shigella sonnei*] | | |
|  | + |  |  | AGI19337 | | |
| O66-16 | *aqpZ* | 233 | 55.1 | MULTISPECIES: aquaporin Z | 99/99(232) | |
|  | (20329-21030) |  |  | [*Plesiomonas*] | | |
|  | + |  |  | WP_010862878 | | |

| **Table S11** Characteristics of the ORFs of *P. shigelloides* O75H34 O antigen gene cluster | | | | | | |
| --- | --- | --- | --- | --- | --- | --- |
| **ORF#** | **Gene name /location /direction** | **No. of amino acids** | **G+C content(%)** | **Similar protein/strain/GenBank accession No.** | **% Identity/% Similarity (No. of aa overlap)** | |
| O75-01 | *rep* | 676 | 55.0 | DNA helicase Rep | 100/100(676) | |
|  | (1-2031) |  |  | [*Plesiomonas shigelloides*] | | |
|  | + |  |  | WP_010862901 | | |
| O75-02 | *rfaH* | 138 | 51.8 | transcription/translation regulatory transformer protein RfaH | 98/100(138) | |
|  | (2185-2601) |  |  | [*Plesiomonas shigelloides*] | | |
|  | - |  |  | WP_039046405 | | |
| O75-03 | *orf3* | 38 | 56.0 | No significant similarity found |  | |
|  | (2705-2821) |  |  |  | | |
|  | - |  |  |  | | |
| O75-04 | *c5* | 141 | 56.2 | cytochrome c5 | 97/98(139) | |
|  | (2947-3372) |  |  | [*Plesiomonas shigelloides 302-73*] | | |
|  | + |  |  | EON89259 | | |
| O75-05 | *wzz* | 340 | 36.9 | Wzz | 38/54(188) | |
|  | (4004-5026) |  |  | [*Plesiomonas shigelloides*] | | |
|  | + |  |  | [AAG17407](https://www.ncbi.nlm.nih.gov/protein/10442654?report=genbank&log$=protalign&blast_rank=25&RID=1HUPUX1X014) | | |
| O75-06 | *wblB* | 346 | 43.7 | WblB protein | 82/92(321) | |
|  | (5049-6089) |  |  | [*Vibrio albensis VL426*] | | |
|  | + |  |  | EEO03407 | | |
| O75-07 | *vipA* | 424 | 38.3 | Vi polysaccharide biosynthesis protein VipA | 84/90(349) | |
|  | (6111-7385) |  |  | [*Aeromonas veroni*] | | |
|  | + |  |  | AHB32271 | | |
| O75-08 | *glmU* | 145 | 45.3 | N-acetyltransferase | | 92/95(137) |
|  | (7605-8042) |  |  | [*Aeromonas hydrophila*] | | |
|  | *+* |  |  | WP_065476729 | | |
| O75-09 | *wecE* | 358 | 42.5 | DegT/DnrJ/EryC1/StrS family aminotransferase | 87/94(333) | |
|  | (8066-9142) |  |  | [*Vibrio cholerae*] | | |
|  | *+* |  |  | WP_042988074 | | |
| O75-10 | *orf10* | 396 | 49.6 | hypothetical protein BM485_17020 | 35/51(194) | |
|  | (9147-10337) |  |  | [*Desulfobulbaceae bacterium DB1*] | | |
|  | *-* |  |  | OKY73758 | | |
| O75-11 | *wbpC* | 615 | 53.1 | WbpC | 41/58(361) | |
|  | (10334-12181) |  |  | [*Pseudomonas aeruginosa PAO1*] | | |
|  | *+* |  |  | [AAC45854](https://www.ncbi.nlm.nih.gov/protein/1545849?report=genbank&log$=protalign&blast_rank=65&RID=1G1H9UFT01R) | | |
| O75-12 | *wzx* | 389 | 56.4 | O-antigen translocase | 42/63(246) | |
|  | (12417-13586) |  |  | [*Pseudomonas sp. BAY1663*] | | |
|  | *+* |  |  | [EXF44008](https://www.ncbi.nlm.nih.gov/protein/588310108?report=genbank&log$=protalign&blast_rank=2&RID=1HUPUX1X014) | | |
| O75-13 | *wzy* | 430 | 42.9 | B-band O-antigen polymerase | 34/54(234) | |
|  | (13589-14881) |  |  | [*Pseudomonas sp. BAY1663*] | | |
|  | *+* |  |  | [EXF44011](https://www.ncbi.nlm.nih.gov/protein/588310111?report=genbank&log$=protalign&blast_rank=4&RID=1HUPUX1X014) | | |
| O75-14 | *hisH* | 46 | 34.1 | imidazole glycerol phosphate synthase subunit HisH | 74/84(39) | |
|  | (15366-15506) |  |  | [*Xenorhabdus nematophila*] | | |
|  | *+* |  |  | [WP_010847581](https://www.ncbi.nlm.nih.gov/protein/498912381?report=genbank&log$=protalign&blast_rank=1&RID=1G1H9UFT01R) | | |
| O75-15 | *hisF* | 254 | 38.2 | imidazole glycerol phosphate synthase subunit HisF | 87/94(240) | |
|  | (15500-16264) |  |  | [*Vibrio cholerae*] | | |
|  | *-* |  |  | WP_069647659 | | |
| O75-16 | *wbpG* | 368 | 48.7 | LPS biosynthesis protein WbpG | 60/79/(291) | |
|  | (16310-17416) |  |  | [*Pseudomonas sp. BAY1663*] | | |
|  | - |  |  | [EXF44012](https://www.ncbi.nlm.nih.gov/protein/588310112?report=genbank&log$=protalign&blast_rank=26&RID=1G1H9UFT01R) | | |
| O75-17 | *GT* | 371 | 42.3 | glycosyltransferase | 87/93(342) | |
|  | (17397-18512) |  |  | [*Vibrio ordalii*] | | |
|  | *+* |  |  | [WP_010317085](https://www.ncbi.nlm.nih.gov/protein/498002929?report=genbank&log$=protalign&blast_rank=1&RID=1HUPUX1X014) | | |
| O75-18 | *mnaA* | 353 | 47.6 | UDP-N-acetylglucosamine 2-epimerase (non-hydrolyzing) | 89/92(325) | |
|  | (18524-19585) |  |  | [*Vibrio cholerae*] | | |
|  | *-* |  |  | [WP_069647662](https://www.ncbi.nlm.nih.gov/protein/1069258213?report=genbank&log$=protalign&blast_rank=1&RID=1HUPUX1X014) | | |
| O75-19 | *WbuB* | 404 | 37.4 | glycosyltransferaseWbuB | 55/75(206) | |
|  | (19614-20828) |  |  | [*Photorhabdus luminescens*] | | |
|  | *+* |  |  | WP_065388901 | | |
| O75-20 | *wbgX* | 383 | 37.4 | WbgX | 97/98(376) | |
|  | (20830-21981) |  |  | [*Plesiomonas shigelloides*] | | |
|  | *+* |  |  | AAG17414 | | |
| O75-21 | *wbgZ* | 496 | 44.3 | wbgZ | 98/99(492) | |
|  | (23032-24522) |  |  | [*Shigella sonnei*] | | |
|  | *+* |  |  | AGI19337 | | |
| O75-22 | *aqpZ* | 233 | 54.6 | aquaporin Z | 99/100(233) | |
|  | (24641-25342) |  |  | [*Plesiomonas shigelloides*] | | |
|  | *+* |  |  | WP_039046419 | | |

| **Table S12** Characteristics of the ORFs of *P. shigelloides* O76H39 O antigen gene cluster | | | | | | |
| --- | --- | --- | --- | --- | --- | --- |
| **ORF#** | **Gene name /location /direction** | **No. of amino acids** | **G+C content(%)** | **Similar protein/strain/GenBank accession No.** | **% Identity/% Similarity (No. of aa overlap)** | |
| O76-01 | *rep* | 657 | 55.0 | DNA helicase Rep | 99/100(657) | |
|  | (1-1974) |  |  | [*Plesiomonas shigelloides*] | | |
|  | + |  |  | WP_010862901 | | |
| O76-02 | *rfaH* | 136 | 51.8 | transcription/translation regulatory transformer protein RfaH | 96/98(134) | |
|  | (2129-2539) |  |  | [*Plesiomonas shigelloides*] | | |
|  | - |  |  | WP_039046405 | | |
| O76-03 | *c5* | 108 | 56.2 | cytochrome c5 | 99/99(107) | |
|  | (2895-3221) |  |  | [*Plesiomonas shigelloides 302-73*] | | |
|  | - |  |  | EON89259 | | |
| O76-04 | *wzz* | 369 | 38.9 | Wzz | 92/95(346) | |
|  | (3861-4970) |  |  | [*Plesiomonas shigelloides*] | | |
|  | + |  |  | AAG17407 | | |
| O76-05 | *rmlB* | 141 | 56.5 | RmlB | 84/92(131) | |
|  | (5865-6290) |  |  | [*Aeromonas piscicola*] | | |
|  | + |  |  | [ABX39496](https://www.ncbi.nlm.nih.gov/protein/160425304?report=genbank&log$=protalign&blast_rank=16&RID=1G2T1G8N01R) | | |
| O76-06 | *degT* | 358 | 34.9 | DegT/DnrJ/EryC1/StrS family aminotransferase | 71/86(309) | |
|  | (7159-8235) |  |  | [*Shewanella oneidensis*] | | |
|  | + |  |  | WP_011073073 | | |
| O76-07 | *orf7* | 314 | 32.4 | hypothetical protein | 50/69(214) | |
|  | (8965-9909) |  |  | [*Vibrio cyclitrophicus*] | | |
|  | + |  |  | [WP_016797290](https://www.ncbi.nlm.nih.gov/protein/515168790?report=genbank&log$=protalign&blast_rank=3&RID=1G2T1G8N01R) | | |
| O76-08 | *wzx* | 501 | 31.8 | Wzx | | 51/70(346) |
|  | (10364-11869) |  |  | [*Acinetobacter baumannii*] | | |
|  | + |  |  | [ARR95885](https://www.ncbi.nlm.nih.gov/protein/1194457716?report=genbank&log$=protalign&blast_rank=55&RID=1G2T1G8N01R) | | |
| O76-09 | *GT* | 313 | 36.1 | glycosyltransferase family 2 protein | 81/90(284) | |
|  | (11866-12807) |  |  | [*Pseudomonas oleovorans*] | | |
|  | + |  |  | WP_074859194 | | |
| O76-10 | *wzy* | 400 | 31.2 | O121 family O-antigen flippase | 45/69(269) | |
|  | (12818-14020) |  |  | [*Escherichia coli*] | | |
|  | + |  |  | [WP_069906915](https://www.ncbi.nlm.nih.gov/protein/1071963854?report=genbank&log$=protalign&blast_rank=2&RID=1HWDMSBE015) | | |
| O76-11 | *wbpS* | 627 | 49.4 | asparagine synthetase (WbpS) | 64/79(448) | |
|  | (14020-15903) |  |  | [*Pseudomonas poae RE*1-1-14*] | | |
|  | + |  |  | [AGE25017](https://www.ncbi.nlm.nih.gov/protein/445199808?report=genbank&log$=protalign&blast_rank=79&RID=1G2T1G8N01R) | | |
| O76-12 | *GT* | 359 | 45.9 | glycosyl transferase | 91/94(337) | |
|  | (15951-17030) |  |  | [*Vibrio cholerae*] | | |
|  | + |  |  | WP_050541310 | | |
| O76-13 | *vipA* | 422 | 46.0 | Vi polysaccharide biosynthesis UDP-N-acetylglucosamine C-6 dehydrogenase TviB | 92/95(402) | |
|  | (17076-18344) |  |  | [*Plesiomonas shigelloides*] | | |
|  | + |  |  | WP_084977029 | | |
| O76-14 | *vipB* | 330 | 46.1 | Vi polysaccharide biosynthesis UDP-N-acetylglucosaminuronic acid C-4 epimerase TviC | 90/93(309) | |
|  | (18415-19407) |  |  | [*Plesiomonas shigelloides*] | | |
|  | + |  |  | WP_084977028 | | |
| O76-15 | *GT* | 347 | 42.0 | glycosyltransferase family 1 protein | 91/94(326) | |
|  | (19507-20550) |  |  | [*Vibrio cholerae*] | | |
|  | + |  |  | [WP_001188927](https://www.ncbi.nlm.nih.gov/protein/447111671?report=genbank&log$=protalign&blast_rank=1&RID=1HWDMSBE015) | | |
| O76-16 | *wbhp* | 321 | 42.2 | NAD-dependent epimerase/dehydratase | 67/78/(251) | |
|  | (20547-21512) |  |  | [*Shewanella baltica OS155*] | | |
|  | + |  |  | [ABN62361](https://www.ncbi.nlm.nih.gov/protein/125998286?report=genbank&log$=protalign&blast_rank=27&RID=1HWDMSBE015) | | |
| O76-17 | *wbgZ* | 420 | 44.3 | wbgZ | 97/98(415) | |
|  | (22816-24078) |  |  | [*Shigella sonnei*] | | |
|  | + |  |  | AGI19337 | | |
| O76-18 | *aqpZ* | 233 | 54.6 | aquaporin Z | 99/100(233) | |
|  | (24197-24898) |  |  | [*Plesiomonas shigelloides*] | | |
|  | + |  |  | WP_039046419 | | |
